# Attentive brain states in infants with and without later autism

**DOI:** 10.1038/s41398-021-01315-9

**Published:** 2021-03-30

**Authors:** Anna Gui, Giorgia Bussu, Charlotte Tye, Mayada Elsabbagh, Greg Pasco, Tony Charman, Mark H. Johnson, Emily J. H. Jones

**Affiliations:** 1 Centre for Brain and Cognitive Development, Birkbeck College, University of London, Malet Street, London, WC1E 7HX UK; 2 Department of Cognitive Neuroscience, Donders Institute for Brain, Cognition and Behaviour, Radboud University Medical Center, Kapittelweg 29, 6525 EN Nijmegen, The Netherlands; 3 Department of Child & Adolescent Psychiatry & Department of Psychology, King’s College London, De Crespigny Park, London, SE5 8AF UK; 4 Montreal Neurological Institute, McGill University, 3801 Rue University, Montréal, QC H3A 2B4 Canada; 5 Department of Psychology, Institute of Psychiatry, Psychology & Neuroscience, King’s College London, De Crespigny Park, London, SE5 8AF UK; 6 Department of Psychology, Cambridge University, Downing Street, Cambridge, CB2 3EB UK

**Keywords:** Neuroscience, Human behaviour

## Abstract

Early difficulties in engaging attentive brain states in social settings could affect learning and have cascading effects on social development. We investigated this possibility using multichannel electroencephalography during a face/non-face paradigm in 8-month-old infants with (FH, *n* = 91) and without (noFH, *n* = 40) a family history of autism spectrum disorder (ASD). An event-related potential component reflecting attention engagement, the Nc, was compared between FH infants who received a diagnosis of ASD at 3 years of age (FH-ASD; *n* = 19), FH infants who did not (FH-noASD; *n* = 72) and noFH infants (who also did not, hereafter noFH-noASD; *n* = 40). ‘Prototypical’ microstates during social attention were extracted from the noFH-noASD group and examined in relation to later categorical and dimensional outcome. Machine-learning was used to identify the microstate features that best predicted ASD and social adaptive skills at three years. Results suggested that whilst measures of brain state timing were related to categorical ASD outcome, brain state strength was related to dimensional measures of social functioning. Specifically, the FH-ASD group showed shorter Nc latency relative to other groups, and duration of the attentive microstate responses to faces was informative for categorical outcome prediction. Reduced Nc amplitude difference between faces with direct gaze and a non-social control stimulus and strength of the attentive microstate to faces contributed to the prediction of dimensional variation in social skills. Taken together, this provides consistent evidence that atypical attention engagement precedes the emergence of difficulties in socialization and indicates that using the spatio-temporal characteristics of whole-brain activation to define brain states in infancy provides an important new approach to understanding of the neurodevelopmental mechanisms that lead to ASD.

## Introduction

Autism spectrum disorder (ASD) affects between 1 and 2% of the population in Western countries^1–3^ but little is known about common mechanisms leading to symptomatology^4^. ASD is a neurodevelopmental condition defined by difficulties in social communication and interaction, and the presence of restricted and repetitive patterns of behaviour and sensory difficulties that emerge during childhood^5^. Although ASD is highly heritable, community diagnosis is not typically made until age 4 or older^6^, limiting possibilities for early intervention. Prospective longitudinal studies of brain development beginning from infancy provide promising opportunities to study the emergence of neurodevelopmental disorders^7,8^. Progress requires revealing the developmental processes that canalise the diverse set of identified genetic and environmental risk factors^9^ towards a coherent phenotypic profile that can be reliably recognized at the categorical level by trained clinicians^10,11^. In this study, we focus on a candidate process that might be involved in the pathway to ASD traits: engagement of attentive brain states in response to social stimuli.

Infants with an older sibling with ASD have an elevated-likelihood of developing ASD themselves, as the prevalence of ASD in this population is 20 times higher than in the typical population^12^. Perspective longitudinal studies of infants with a family history of ASD (also called high-risk or elevated-likelihood infants) show that behavioural differences in social attention emerge over the first two years of life. For example, Ozonoff and colleagues describe a declining trajectory of looking to faces between 6 months and 3 years in infants with emerging ASD^13^. More fine-grained measures of visual attention indicate that infants later diagnosed with ASD show declining attention to the eyes in social videos between 2 and 6 months^14^, and at 6 months shorter epochs of attention to faces^15^, and less attention to videos of women^16^, particularly when they are talking^17^. In a temporally-resolved eye-tracking analysis, 10-month-old infants with a family history of ASD tend to look less towards faces from 300 ms after the adult initiated direct gaze during naturalistic interactions; relation to later ASD outcome was not tested^18^. Taken together, it appears that attention to important features of social interaction (like faces with direct gaze) may be altered in the emergence of ASD.

One leading hypothesis suggests that failure to engage attentive brain states when interacting with people might disrupt the experience-dependent development of social cognition in ASD^11,19,20^. Indeed, in toddlers with ASD altered neural attention responses to faces relate to broader delays in socialization skills^21^, and stronger brain responses to faces are associated with an improvement of social symptoms following behavioural intervention^22^. If disrupted cortical social attention is involved in the pathway to ASD, atypical neural responses to people should be seen between 6 and 12 months, when particular brain areas or networks become increasingly tuned to respond to social cues^23^. Indeed, prospective studies have shown reduced cortical responses to social videos at 4 to 6 months^23^, reduced neural sensitivity to gaze shifts at 6–9 months^24^, and altered neural responses to faces vs objects^15^ in infants with a later ASD diagnosis. Interestingly, around 10 months of age, infants with a family history of ASD show slower brain responses to faces with a direct vs averted gaze^25^ and slower and reduced brain response to the gaze shifting towards vs away from them^24^, possibly indicating a link between ASD liability and early neural sensitivity to eye gaze. However, it is still unclear whether these findings reflect different neural attention engagement depending on face presence and gaze direction, and whether this is linked to ASD symptomatology in the child and in the family. In the present study, we built on these preliminary signals to examine neural correlates of attentional engagement to faces with direct and averted gaze and a control non-face stimulus in a larger cohort of infants with and without family history of ASD.

Attention engagement in the infant brain has been primarily explored using event-related potentials (ERPs). The Nc (‘negative central’) component is a negative deflection measured around 300 ms after stimulus onset over frontal regions in infancy^26^. Studies manipulating stimulus characteristics and including concurrent arousal measures have consistently shown that this ERP component reflects engagement of attention to interesting or salient stimuli^27–31^. One small previous study in infants with later ASD (*n* = 6) reported a smaller (less negative) mean amplitude and shorter latency of the Nc when attending to faces compared with typically developing infants (*n* = 25)^15^. Moreover, it has been suggested that atypical topography of the Nc (reduced responses over the right frontal region) is observed as an early sign of ASD^21^. However, such traditional approaches to ERP analysis depend on a priori selection of regions of interest thought to reflect the activity of particular underlying neural populations^32^. This approach increasingly contrasts with recent shifts to view attention as reflecting a state of the brain spread over a broad distributed network^27,33–37^, requiring new analytic methods to be employed.

In the present study, we combined traditional top-down specified approaches and data-driven discovery methods to study attentive brain states to faces with direct and averted gaze vs a control stimulus in a larger sample of infants with (*n* = 19) and without (*n* = 112) later ASD. First, we attempted to replicate the previous observation of a reduced Nc response to faces vs non-social stimuli^15^. Power analysis conducted with G*Power 3.1^38^ based on the effect size of the previous study (*η*^2^ = 0.17 for Nc mean amplitude) showed that a power of 0.80 to find a significant interaction between group and stimulus at a *p*-value of 0.05 would have been obtained with groups of at least 11 individuals, confirming that our sample size was adequate. We used a classic statistical approach to examine whether Nc features were associated with ASD liability at a group level as well as dimensional variation in social skills. Second, we conducted fine-grained analyses of the spatio-temporal characteristics of the entire scalp field topography, to see whether infants with later ASD showed atypical transient brain states (microstates) when attending to faces with direct or averted gaze and a control stimulus. Since hypothesis-driven approaches can miss other important contributors to variance, we triangulated the top-down analysis approach by using a genetic algorithm and elastic-net regularization on microstate features across the set of stimuli presented to identify those that were most predictive of later ASD and socialization skills at the individual level. This allowed us to test whether our theory-informed selection of the neural correlates of social attention engagement was consistent with a data-driven approach to identifying the most informative brain responses with respect to later ASD diagnosis and symptomatology^39^. We examined the relation between attentive brain states and both categorical outcome of ASD, and dimensional variation in social adaptive skill, in line with recent evidence that the causative factors underlying diagnosis of ASD may be separable from those underlying dimensional variation in relevant symptom domains^40^.

## Materials and methods

### Participants

Participants were recruited through the British Autism Study of Infant Siblings (BASIS, www.basisnetwork.org), a longitudinal study of infants with older siblings with and without ASD. As part of Phase 1 and Phase 2 of this study, 170 infants with a family history of ASD (FH, referred to as high-risk or elevated-likelihood infants in other research) were enrolled in the first year of life and followed to age 3 years. At enrolment, all children in the FH group had an older sibling who received a diagnosis of ASD from a UK clinician. Additionally, a control group of 77 infants with at least one older sibling and no history of ASD in first- and second-degree relatives (noFH, also called low-risk or typical-likelihood) followed a parallel assessment protocol. Developmental level was assessed at each visit using the Mullen Scales of Early Learning (MSEL)^41^. At 36 months all participants were assessed using the Autism Diagnostic Observation Schedule-Generic (ADOS-G)^42^. Experienced researchers, informed by their observations and outcomes from the ADOS-G, MSEL and the Autism Diagnostic Interview-Revised (ADI-R)^43^ administered to parents, determined whether each child did (FH-ASD) or did not (FH-noASD) meet best estimate research diagnosis of DSM-5 ASD criteria. None of the noFH children met criteria for an ASD research diagnosis nor had received a community diagnosis of ASD by 3 years of age (hereafter, noFH-noASD). Overall MSEL, VABS and ADOS scores of the noFH-noASD children at 3 years fell within the typical range (see Table 1). The Supplementary Materials section SM1 provides details on the entire protocol and group assignment criteria.

**Table 1:** Demographic characteristics and scores of the behavioural measures of the participants who provided data for the present study, divided into outcome groups.

	noFH-noASD	FH-noASD	FH-ASD		
N current study	40	72	19		
Phase (1/2)	31/9	22/50	9/10		
Sex (M/F)	16/24	34/38	15/4		
**Participants**	**Mean (s.d.) min–max**	**Mean (s.d.) min–max**	**Mean (s.d.) min–max**	***p*-value**	${\mathbf{\it{\eta}}}^{\mathbf{2}}_{\mathbf{\it{P}}}$
Age (days)	244.97 (40.65) 6–11	261.33 (36.66) 6–11	251.21 (31.64) 6–10	0.079	0.04
*8 months*					
MSEL Composite score	106.33 (11.54) 86–132	103.15 (15.09) 70–134	100.17 (16.75) 77–139	0.285	0.02
VABS Composite score	100.67 (12.74)^a^ 78–130	93.51 (13.53)^a^ 66–150	92.72 (10.83) 71–113	0.015*	0.07
VABS Socialization score	103.23 (12.78) 81–132	98.85 (12.97) 70–152	98.22 (10.03) 81–118	0.175	0.03
VABS Communication score	101.88 (13.03)^a^ 66–123	94.75 (16.77)^a^ 55–143	94.84 (11.51) 70–112	0.048*	0.05
VABS Daily Living skills score	100.55 (15.25) 54–122	100.79 (13.63) 54–143	97.74 (13.51) 77–117	0.697	0.01
VABS Motor skills score	97.45 (14.11)^a,b^ 73–127	85.58 (16.19)^a^ 56–144	84.16 (13.69)^b^ 56–106	<0.001*	0.12
*3 years*					
MSEL Composite score	115.50 (15.06)^a^ 80–147	108.35 (20.61)^b^ 63–145	92.39 (26.19)^a,b^ 49–142	0.001*	0.12
VABS Composite score	107.26 (9.17)^a,b^ 93–131	99.06 (9.15)^a,c^ 78–121	82.05 (11.74)^,b,c^ 57–100	<0.001*	0.41
VABS Socialization score	105.79 (7.11)^a,b^ 94–122	99.21 (9.51)^a,c^ 72–116	78.42 (12.49)^b,c^ 61–110	<0.001*	0.47
VABS Communication score	107.94 (11.05)^a, b^ 85–139	100.89 (10.79)^a,c^ 76–125	87.83 (15.12)^b,c^ 52–112	<0.001*	0.24
VABS Daily living skills score	70.76 (15.86)^a,b^ 27–96	60.54 (20.56)^a,c^ 16–95	27.00 (23.91)^b,c^ 1–90	<0.001*	0.34
VABS Motor skills score	101.65 (13.22)^a,b^ 61–124	93.33 (10.93)^a,c^ 70–124	85.26 (10.78)^b,c^ 64–100	<0.001*	0.12
ADOS-2 CSS	2.50 (1.86)^a^ 1–7	2.70 (2.12)^b^ 1–8	5.47 (2.99)^a,b^ 1–10	<0.001*	0.18

*noFH-noASD* no family history of autism spectrum disorder (ASD) and no diagnosis at 3 years, *FH-noASD* family history without a diagnosis of ASD at 3 years, *FH-ASD* family history of ASD who received a diagnosis of ASD at 3 years, *MSEL* Mullen Scales of Early Learning, *VABS* Vineland Adaptive Behaviour Scales, *ADOS2-CSS* autism diagnostic observation schedule, 2^nd^ edition, with calibrated severity scores calculated as explained in SM1.

N: number of subjects with available scores; *s.d.*: standard deviation; *p*-value of the one-way ANOVA with outcome groups as between-subjects factor, for age, MSEL and VABS scores, and Kruskal–Wallis non-parametric test for ADOS scores.

${\it{\upeta }}_p^2$: partial eta-squared as a measure of the effect size.

^a,b,c^Superscript letters denote that groups are significantly different from each other based on Tukey’s Honest Significant Difference post-hoc analyses with 95% family-wise confidence level for age, MSEL and VABS scores, and based on pairwise comparisons using Mann–Whitney *U* test with Bonferroni correction for multiple comparisons for ADOS-2 CSS.

*p < 0.05.

EEG and behavioural data were collected between 6 and 11 months from 247 infants (*M* = 7.92, SD = 1.26). Of these, 131 infants provided sufficient EEG and behavioural data (40 noFH-noASD, 72 FH-noASD, 19 FH-ASD; see Table 1, Supplementary Table S1 and Supplementary Fig. S1). Ethical approval for BASIS Phase 1 and 2 data collection was obtained from NHS Health Research Authority (REC reference number 08/H0718/76 and 06/MRE02/73). Informed consent was obtained from parents of all the infants taking part in the study.

### Electrophysiological recording and processing

The task and procedure have been described previously in a study investigating different ERP components on a partly overlapping cohort (Phase 1, *n* = 62)^24^. Infants sat on their parent’s laps 60-cm from a 40 × 29-cm computer screen while brain activity was continuously recorded with a 128-channel Hydrocel Sensor Net at a sampling rate of 500 Hz. 50 blocks were presented continuously for as long as the child remained attentive. Each block started with a static colourful fixation stimulus presented for a variable duration of 800–1200 ms, followed by a colour picture of a female model whose gaze was directed either toward (face with direct gaze, or FD, Fig. S2A) or away (face with averted gaze, or FA, Fig. S2B) from the infant; gaze then shifted towards or away for three to six times (not analysed in the present study). Faces were presented in a pseudorandom order. Additionally, approximately one-third of the blocks consisted in the non-social control stimuli (‘Noise’, Fig. S2C). The trial duration was 800 ms, followed by a 500-ms interval with no visual stimulus. Trials were included in the electrophysiological data analysis only if the infants were fixating the centre of the screen at target onset, without any gaze shifts, blinking or head movements during the 800 ms following stimulus onset, identified through offline video coding. Infants were included if they provided at least 10 minimal-artifact trials in each condition. Table S2 summarises the number or valid trials in each condition per group. Data were stored and analysed offline in EGI NetStation 4 (for Phase 1) and 5 (for Phase 2) using the same protocol as ref. ^24,44^. Full details on the EEG data pre-processing are described in SM2.

### Analyses

We examined the relation between infant brain activity and later behaviour at two different levels: classic ERP analyses and data-driven microstate analyses using machine learning. We looked at whether the early brain measures were associated with (1) ASD liability, examining group differences based on ASD family history and clinical outcome defined at three years of age (noFH-noASD vs FH-noASD vs FH-ASD), and (2) dimensional variation in social skills, i.e., later social behaviour as assessed using the VABS Socialization standard scores at 3 years of age. This measure was selected as a dimensional measure of social adaptive skills with minimal skew and associated with genetic variation^45^. Of note, VABS Socialization scores was preferred over measures of social symptom load (like the ADOS) as it better captures variation across infants with and without a diagnosis. For example, the social affect score of the ADOS or the social communication impairment (SCI) T-score of the social responsiveness scale (SRS), showed very skewed distributions (Fig. S3).

#### Classic event-related potentials

**Fig. 1:**
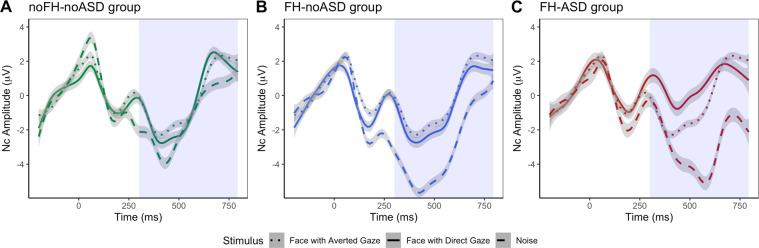
Anterior event-related potentials for the three ASD liability groups. Illustration of the grand average ERPs over the frontal electrodes at 8 months for the noFH-noASD (**A**), FH-noASD (**B**) and FH-ASD (**C**) groups, with shaded area highlighting the Nc time window (x-axis, 300–800 ms). ERP data have been smoothed for representation purposes using the ‘gam’ function of the ‘ggplot2’ package in R^92^.

Following previous research^15,21^, Nc amplitude was defined as the mean amplitude of the negative deflection between 300 and 800 ms after stimulus onset across left, central and right frontal regions (Fig. S4). Nc mean amplitude and peak latency (the most negative point within the same time-window) in response to FD, FA and Noise were extracted using the ‘erp.easy’ R-package and individually verified through visual inspection. Figure 1 depicts the ERP component in response to the FD, FA and Noise stimuli, averaged across the three frontal regions, for the three ASD liability groups.

For the categorical analyses testing differences between ASD liability groups, linear mixed-effects models (‘lme’ function of the ‘lmne’ package in R^46^) were used, with Nc mean amplitude and latency as dependent variables, respectively. We tested for the fixed effects of region (right, central, left), group (noFH-noASD, FH-noASD, FH-ASD), stimulus (FD, FA and Noise) and the interaction between group and stimulus, with participant as random effect nested within region and stimulus. Age (184–351 days), sex (female, male), and developmental level (measured with the MSEL Early Learning Composite at 8 months) were included as covariates in the baseline model. Model fit was tested using maximum likelihood^47^. Within the model with the lowest Akaike information criterion value^48,49^, we further examined significant effects based on planned contrasts, defined to investigate:

- group differences: (1) family history; noFH-noASD vs FH-noASD + FH-ASD, and (2) ASD outcome; noFH-noASD + FH-noASD vs FH-ASD;
- stimulus differences: (1) social content; FD + FA vs Noise, and (2) gaze direction; FD vs FA.

To further investigate significant differences between stimuli by group, we run repeated measures-ANOVAs and Tukey honestly significant difference (HSD) post-hoc tests. Generalized eta squared ($\eta _2^G$) was used as a measure of the effect size, as recommended for repeated measures designs^50^.

For dimensional analyses, two linear regressions were used, with VABS Socialization scores at 3 years as dependent variable and either Nc mean amplitude or latency difference scores between FD and Noise (selected to reflect social-specific aspects of attention with the strongest ASD-related contrast, based on the results of our categorical analyses) as independent variable. Sex, age (in days) and developmental level were included as covariates. The total number of participants for this analysis was 123, as VABS Socialization scores were not available for 8 participants. In order to verify the specificity of the significant relationship for social skills, we tested the association with a different domain of the same questionnaire, which is not directly influenced by ASD social symptoms (VABS Motor Skills). We also examined the association between VABS scores at three years of age and Nc mean amplitude or latency difference scores between FA and Noise, to see whether effects were dependent on gaze direction.

#### Data-driven microstates

**Fig. 2:**
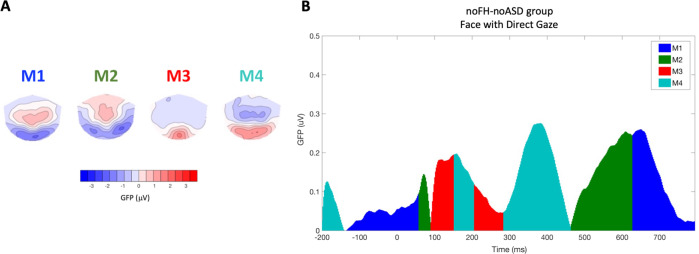
The ‘prototypical’ microstates during social attention. **A** Scalp field topography of the four optimal microstate maps estimated from infants with no Family History without a diagnosis of ASD at three years (noFH-noASD) in response to the face with direct gaze stimulus. Normalised amplitude (GFP) in the microstate ranges from −3.5 (blue) to 3.5 (red) microvolts. **B** Sequence of microstates in response to the face with direct gaze stimulus between −200 and 794 milliseconds (on the *x*-axis). The blue area indicates that the topography of the scalp field reflects microstate map 1 (M1), green reflects microstate map 2 (M2), red reflects microstate map 3 (M3) and cyan reflects microstate map 4 (M4). On the *y*-axis, absolute values of the mean GFP for each time-stamp, in microvolts, are indicated.

Periods of stable topographies of scalp electromagnetic fields of the ERP data, called microstates, were clustered using a randomization-based procedure^51^. As we were interested in ‘prototypical’ microstate maps associated with social processing, we first identified maps in the noFH-noASD group in the FD condition (Fig. 2). Fig. S5 illustrates the procedural steps of the microstate analysis. Microstates cross-validation was performed by Randomization Graphical User interface (RAGU)^52^ using the AACH cross-validation algorithm in the time-window between 0 and 794 ms (see SM3). The optimal number of microstate maps that significantly improved the amount of explained variance in the ERP data was four (Fig. S6 and Table S3). Subsequently, we examined whether there were differences in the degree to which these ‘prototypical’ brain states were expressed in relation to ASD and dimensional variation of social skills. To do this, within RAGU the 4 ’prototypical’ maps were used as templates and fitted (through spatial correlation) to the individual grand-averaged time-locked event-related EEG data^32^ for the other two conditions (FA and Noise) for the noFH-noASD infants and for FD, FA and Noise for the FH infants. As a result of this process, the multichannel evoked potential measurement (represented by a factor vector of the Global Field Power-normalised values at each channel^53^) at each moment in time was classified as belonging to one of the microstate maps^54^.

Subsequently, we used a data-driven analytic approach based on machine-learning algorithms to detect the microstate features most associated with later ASD within the FH group and to dimensional variation of social skills in the entire sample. We selected this approach to reflect the fact that our top-down analysis may have masked the presence of other effects in the data, leading us to an erroneously specific conclusion. Of note, individual-level prediction of categorical diagnosis was considered within the FH group only because no ASD cases were observed in the noFH group and thus include all infants in the analysis would have confounded family history and outcome effects. This was not the case for dimensional measures.

For all microstates, we extracted the duration and mean Global Field Power (GFP, a measure of the strength of the scalp field^32^) in response to FD, FA and Noise for each participant in the Nc time window, between 300 and 794 ms (see SM3). These two features of the microstates were selected as they capture both timing and strength of the brain states, which conceptually map to the traditional latency and amplitude measures used in previous ERP research and the current study.

For prediction of ASD, we performed feature selection using a genetic algorithm to extract information about the most relevant features for classification of ASD outcome in the FH infants (*n* = 91, SM4.a). The sample was split into a main sample (70% of the entire sample, *n* = 64) for model selection, and a separate holdout sample (30% of the entire sample, *n* = 27) for validation. The sample partition was stratified for binary outcome (i.e., FH-ASD vs FH-noASD). A total of 21 variables were used as features, including sex, age (in days), developmental level (MSEL Early Learning Composite at 8 months) and duration and GFP for M1, M2 and M4 in response to each condition (FD, FA, noise). M3 was not included because it was only found in a subset of the infants (n_FH_ = 79; n_FH-noASD_ = 62, n_FH-ASD_ = 17). Developmental level was included in this analysis to observe to what extent attentive brain states predicted individual outcome compared with a standardized behavioural assessment of cognitive skills. Fitness, or predictive accuracy of each model, was measured by the area under the curve (AUC) of a 10-fold cross-validated support vector machine (SVM) classifier built on the set of features under evaluation. The feature set providing the highest AUC (> 75%) in the evolutionary process (optimal set) and the features with highest incidence (higher than 80%) were selected as input for the classifier analysis (highest incidence set). Classification performance was tested on the separate holdout sample for classifiers built on (1) the optimal set from feature selection, indicating the set of features that performed best for classification; (2) the set of features with highest incidence (f > 0.8) in the feature sets with highest performance (AUC > 0.75) during repeated evolution of the genetic algorithm, representing the most replicable set of features across repetitions. To evaluate classification performance, we computed AUC, sensitivity, specificity, accuracy, negative predictive power (NPV), and positive predictive power (PPV) from the ROC curve. The final metrics with errors were obtained from the average and standard deviation values over 1000 repetitions of the entire procedure, and the 95% confidence interval of each metric was also averaged over repetitions. We tested for significant difference of the classifier performance (AUC) from chance level, and between classifiers through a shuffle test^55^. Of note, asynchrony in group size was addressed through different cost weights when training the SVM algorithm, so that the misclassification cost was reweighted to take into account unbalanced data.

For prediction of dimensional variation in social skills (VABS Socialization scores at 3 years), regression with elastic-net regularization was used to select relevant microstate features across the entire sample (*n* = 123). The same 21 variables used as features for the classifier analysis were used as predictors. Details on data pre-processing can be found in SM4.b. Leave-one-out cross-validation was used to cross-validate the predictive model, and nested 10-fold cross-validation with 10 repetitions was used for parameter optimization based on minimization of the root mean squared error (RMSE). To evaluate predictive performance, we computed RMSE and the relative error (RMSE/range of outcome scores). 95% confidence interval (CI) for RMSE was computed using bootstrap with 1000 repetitions, while the *p*-value was computed through a shuffle test^55^.

## Results

### Speed and depth of attention engagement: event-related potentials

#### Relation to ASD

EEG data collected from 131 8-month-old infants were used for these group analyses, comparing three ASD liability groups: noFH-noASD, FH-noASD, FH-ASD (see ‘Materials and methods’ and SM1 for details). Based on a previous study^15^, smaller amplitudes and shorter latencies when attending to static faces with direct gaze than to Noise were expected in the FH-ASD infants, suggesting that neural correlates of reduced social attention engagement to features that are important during social interaction precede the development of difficulties in socialization.

##### Nc amplitude

The Nc amplitude data were normally distributed for all groups in the FD (Shapiro–Wilk test noFH-noASD: *W* = 0.978, *p* = 0.601, FH-noASD: *W* = 0.982, *p* = 0.414, FH-ASD: *W* = 0.948, *p* = 0.372), FA (noFH-noASD: *W* = 0.973, *p* = 0.458, FH-noASD: *W* = 0.981, *p* = 0.387, FH-ASD: *W* = 0.953, *p* = 0.437) and Noise condition (noFH-noASD: *W* = 0.968, *p* = 0.307, FH-noASD: *W* = 0.983, *p* = 0.453, FH-ASD: *W* = 0.928, *p* = 0.157). Groups had similar variance for the FD (Levene’s test: *F*(2,128) = 0.355, *p* = 0.702), FA (*F*(2,128) = 0.635, *p* = 0.532) and Noise condition (*F*(2,128) = 0.828, *p* = 0.439).

The linear mixed-model on Nc amplitude by stimulus, region, and group revealed a significant main effect of stimulus (*χ*^2^(14)= 30.882, *p* < 0.001), that varied by both the social content of the stimulus (faces vs Noise: *β* = 0.485, s.e. = 0.184, *p* = 0.008) and also gaze direction (FD vs FA: *β* = −0.770, s.e. = 0.318, *p* = 0.016). There was also a significant stimulus-by-outcome interaction (*χ*^2^(18) = 10.245, *p* = 0.037) with a significant difference between noFH-noASD and FH (FH-noASD + FH-ASD) infants in the effect of gaze direction (*β* = 1.058, s.e. = 0.397, *p* = 0.008). The contrasts testing noFH-noASD + FH-noASD vs FH-ASD were not significant (p*s* > 0.321), indicating that the mixed-model significant interaction effect was related to family history and not ASD outcome. All the model fit results are reported in the Supplementary Materials (Table S4).

**Fig. 3:**
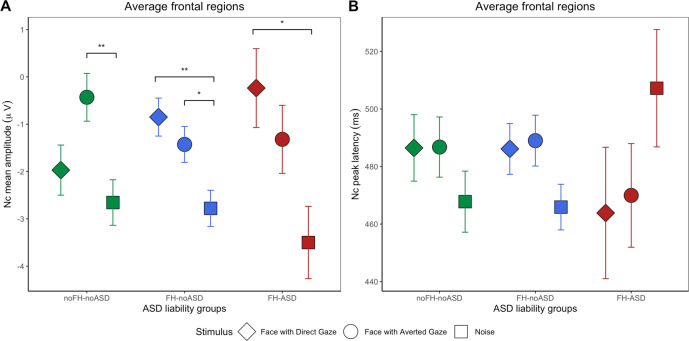
Mean Nc features by group and stimulus. Mean amplitude (**A**) and peak latency **B** of the Nc by stimulus and ASD liability group, averaged across the frontal regions. All error bars represent ± 1 standard error.

Follow-up ANOVAs testing the effect of stimulus in each group revealed that in the noFH-noASD children (*F*(1,238) = 6.323, *p* = 0.002, $\eta _2^G$ = 0.023) there was a significant difference between FA and Noise (*p* = 0.01) but no difference between FD and Noise (*p* = 0.120) nor between FD and FA (*p* = 0.653). In the FH-noASD group, the effect of stimulus (*F*(1,430) = 8.25, *p* < 0.001, $\eta _2^G$ = 0.019) was underpinned by a significantly smaller Nc to FD and to FA compared to Noise (*p* = 0.002 and *p* = 0.044 respectively), with no FD-FA difference (*p* = 0.561). In the FH-ASD infants (*F*(2,142) = 5.109, *p* = 0.007, $\eta _2^G$ = 0.045) there was a smaller Nc to FD than Noise (*p* = 0.021) but no difference between FA and Noise (*p* = 0.214) nor between FD and FA (*p* = 571). These results are illustrated in Fig. 3A.

##### Nc latency

The Nc latency data in response to FD were normally distributed for the noFH-noASD (*W* = 0.960, *p* = 0.167) and FH-ASD (*W* = 0.935, *p* = 0.218) but moderately skewed for the FH-noASD group (*W* = 0.914, *p* = 0.005, skewness=0.962). Nc latency to FA was normally distributed for the FH-ASD group (*W* = 0.940, *p* = 0.270) but moderately skewed for the noFH-noASD (*W* = 0.927, *p* = 0.013, skewness = 0.790) and FH-noASD groups (*W* = 0.930, *p* < 0.001, skewness = 0.893). For the Noise condition, data were normally distributed in the FH-ASD group (*W* = 0.928, *p* = 0.157) but moderately skewed for the noFH-noASD (*W* = 0.914, *p* = 0.005, skewness = 0.816) and FH-noASD groups (*W* = 0.915, *p* < 0.001, skewness = 0.962). Variances were homogeneous between groups for the FD (Levene’s test: *F*(2,128) = 1.49, *p* = 0.229), FA (*F*(2,128) = 0.288, *p* = 0.750) and Noise condition (*F*(2,128) = 2.206, *p* = 0.114).

The linear mixed-model testing the effects of region, group and stimulus on Nc peak latency revealed a significant effect of ASD liability group (*χ*^2^(12) = 6.824, *p* = 0.033). Planned contrasts indicated that infants who did not receive a diagnosis of ASD at three years (noFH-noASD + FH-noASD) had overall longer latencies than FH-ASD infants (*β* = 46.143, s.e. = 17.664, *p* = 0.010). There was also a main effect of region (*χ*^2^(10) = 7.885, *p* = 0.019, Fig. S7) and a nearly significant interaction between stimulus and group (*χ*^2^(18) = 9.224, *p* = 0.056), explained by a significant difference in Nc latency between infants with (FH-ASD) and without (noFH-noASD+FH-noASD) later ASD for the social (FD + FA) vs non-social (Noise) stimulus contrast (*β* = −20.091, s.e. = 6.796, *p* = 0.003) such that, descriptively, shorter latencies to faces than Noise were observed in the FH-ASD group, while longer latencies to faces than Noise were observed in the noFH-noASD and FH-noASD groups. All differences between stimuli were non-significant in follow-up post-hoc tests (p*s* > 0.119). Figure 3B provides a graphical representation of these results. All model fit results can be found on Supplementary Table S5.

#### Relation to VABS Socialization scores

##### Nc amplitude

**Fig. 4:**
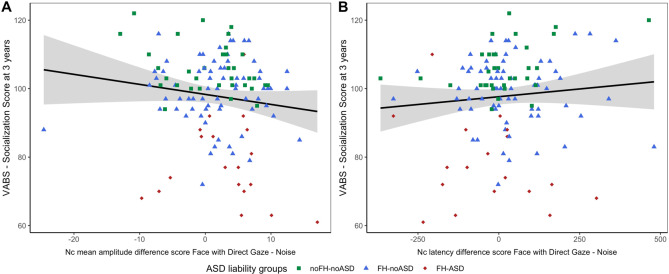
Relationship between Nc features and later dimensional variation in social adaptive skills. Mean amplitude (**A**) and peak latency **B** difference between face with direct gaze and Noise at 8 months, on the *x-*axis.

Across the whole cohort, a relatively larger Nc to FD than Noise (i.e., more negative Nc amplitude difference score between FD and Noise) was associated with higher VABS Socialization scores, indicating better social skills, at 3 years (*β* = −0.350, s.e. = 0.180, *p* = 0.054, Fig. 4A) after controlling for the effect of sex (*β* = 6.377, s.e. = 2.171, *p* = 0.004), age (*β* = −0.049, s.e. = 0.029, *p* = 0.101) and developmental level at 8 months (β = 0.118, s.e. = 0.076, *p* = 0.126). The model met the general assumptions for linear regression: mean of residuals approaching 0 (−7.029 × 10^−16^), no correlation between independent variable and residuals (*r* = −5.95 × 10^−17^, *p* = 1), no autocorrelation of residuals (Durbin–Watson test: DW = 1.841, *p* = 0.174), no multicollinearity (variance inflation factor: VIF = 1.043), although residuals were moderately skewed (skewness = 0.646).

To test for specificity, we additionally examined the association with the motor skills domain of the VABS as a non-social related measure of parent-reported adaptive behaviour, and found no significant association (*β* = −0.117, s.e. = 0.173, *p* = 0.498). The Nc amplitude difference score between FA and noise was not significantly associated with VABS Socialization scores (*β* = −0.298, s.e. = 0.207, *p* = 0.153).

##### Nc latency

There was a trend-level association between latency difference score between FD and Noise and VABS Socialization at three years of age (*β* = 0.014, s.e. = 0.008, *p* = 0.080, Fig. 4B) after controlling for sex (*β* = 6.82, s.e. = 2.193, *p* = 0.002), age (*β* = − 0.035, s.e. = 0.028, *p* = 0.225) and developmental level in infancy (*β* = 0.109, s.e. = 0.077, *p* = 0.159). The regression model generally met the general assumptions for linear regression: mean of residuals = 1.88 × 10^−16^, no correlation between independent variable and residuals (*r* = 4.62 × 10^−18^, *p* = 1), no autocorrelation of residuals (DW = 1.796, *p* = 0.112), no multicollinearity (VIF = 1.015), although residuals were moderately skewed (0.964).

The association between VABS Socialization scores and latency difference score between FA and Noise was non-significant (*β* = 0.001, s.e. = 0.008, *p* = 0.940).

### States of attention: microstates

The results of the top-down analysis were relatively consent with the previous work^15^. However, our a priori selection of regions, features and contrasts between stimuli may have missed important features relevant to ASD. To examine this, we conducted a bottom-up data-driven analysis that broadly characterised states of brain activity and examined which were most informative in predicting later categorical and dimensional traits.

Figure 2 shows the four ‘prototypical’ microstates extracted from the noFH-noASD group in the time window between −200 and 794 ms in the FD condition.

#### Relation to ASD

In the machine-learning analysis, including the duration and global field power (GFP) of each microstate (M1, M2 and M4) from all conditions (FD, FA and Noise) for each infant as well as sex, age and developmental level, frequency analysis on repeated evolution showed that the most relevant features for prediction of ASD clinical outcome (incidence higher than 80%) were: shorter duration of M1 and M4, and longer duration of M2 in response to FD. Using this highest incidence set of features, classification was possible with 62.7% AUC (95% CI; [50.9, 90.0]; *p* = 0.09, Table S6). The classifier showed significant accuracy (*M* = 70.0, 95% CI; [63.6, 90.0]; *p* < 0.0001), specificity (*M* = 100; 95% CI [31.8, 100]; *p* < 0.001) and PPV (*M* = 100; 95% CI [59.5, 100]; *p* < 0.001), but poorer sensitivity (*M* = 40.0; 95% CI [40.0, 100]; *p* = 0.54) and NPV (*M* = 62.5; 95% CI [62.5, 100]; *p* = 0.54). There was no significant difference in classification performance between the optimal and the highest incidence set (*p* = 0.8, Fig. S8). Taken together, this suggests that whilst individual-level prediction remains challenging, the duration of the different microstate responses to faces with direct gaze can identify a proportion of children with later ASD with relative accuracy. However, the sensitivity and specificity profile indicate that not all children with ASD show atypicalities in this domain.

#### Relation to VABS Socialization scores

**Fig. 5:**
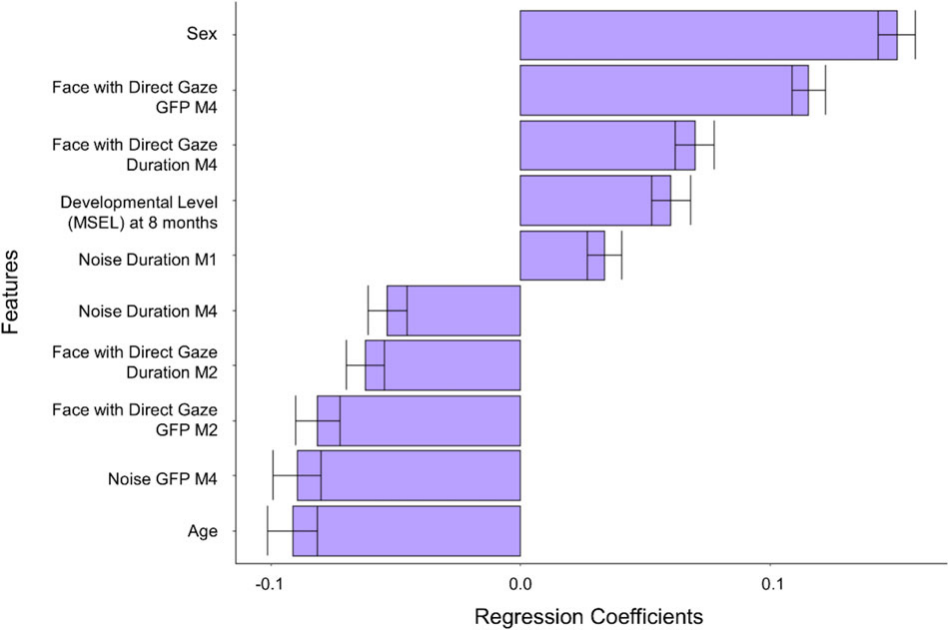
Individual prediction of dimensional variation in social skills. Regression coefficients for prediction of VABS Socialization score at 3 years at an individual level are displayed on the *x*-axis, using demographic data (sex, age in days, developmental level as measured by the MSEL Early Learning Composite score at 8 months) and microstate features in response to faces with direct or averted gaze, and Noise. Microstate features included duration and global field power (GFP) of microstates 1 (M1), 2 (M2) and 4 (M4). Only coefficients that were always selected by the elastic-net regression model with leave-one-out cross-validation are reported. Bars indicate the average of regression coefficients over cross-validation folds is shown, and error bars indicate the standard deviation. Coefficients are in standard units.

On a dimensional level, prediction of social skills at 3 years as measured by VABS Socialization scores was possible with an average RMSE = 12.54 (95% CI; [10.7; 14.2]; *p* = 0.06), corresponding to 20.5% error relative to the score range in the sample (relation between predicted and observed values: *β* = 0.14 (*t*(120) = 1.53, *p* = 0.13). The elastic-net regression model indicated a range of features relevant to prediction depicted in Fig. 5. The microstate values most strongly related to higher VABS Socialization scores at 3 years were: larger GFP and longer duration of M4 in response to FD, and smaller GFP of M4 in response to Noise; these outperformed developmental level at 8 months. Of note, sex had the largest regression coefficient for prediction of later VABS Socialization scores, indicating that males tended to have lower social scores at 3 years.

Thus, the data-driven machine-learning analyses indicated that, at an individual level, duration of M1, M2 and M4 in response to FD were the most reliable predictors of categorical ASD outcome, while GFP of M4 in response to FD and Noise and duration of M4 to Noise were the microstate features with stronger association with dimensional variation in social skills measured with VABS Socialization score at 3 years of age, although the trend of association between the set of features and VABS Socialization scores was only marginally significant.

## Discussion

We found that patterns of attention engagement to faces and non-faces in infancy vary with family history and outcome of ASD, and with difficulties in socialization in childhood. We operationalised attention engagement by examining event-related profiles of neural activity in response to face with direct or averted gaze and non-face (visual noise) stimuli in a relatively large sample of infants with and without older siblings with ASD. Our combined results from hypothesis-driven analysis of the Nc attention-related component and a data-driven examination of states of brain activity support the proposition that infant social attention is related to later social functioning. However, results also suggest that duration and amplitude of neural responses may relate to different aspects of ASD liability; atypically enhanced processing of non-social stimuli is often as informative as reduced processing of social stimuli, altered brain responses to faces with direct gaze contribute more to the prediction of ASD at a categorical and dimensional level than responses to faces with averted gaze, and using data-driven approaches can broaden our understanding of the neurodevelopmental mechanisms leading to ASD.

### Atypical social attention

Both analyses were consistent with the previous work^15^ showing that some elements of social attention are atypical in early ASD. First, we conducted a hypothesis-driven examination of the Nc component (an event-related potential measured over a specific time window and scalp region that has robust previous evidence for links to attention^27–31,56^). Consistent with the pattern of results reported in an independent sample^15^, we observed that infants with an older sibling with ASD showed reduced Nc amplitude to faces with direct gaze and enhanced amplitude to a visual noise control stimulus (suggestive of more attention to non-social than social stimuli) relative to infants without a familial history of ASD. Within the entire sample, larger Nc mean amplitude to visual noise versus faces with direct gaze at 8 months of age (suggestive of diminished social attention to highly salient stimuli) was related to poorer social skills at age 3.

In addition to the classic ERP analysis, we conducted a data-driven fine-grained analysis of the spatio-temporal characteristics of the averaged neural signals. This was the first application of microstate analysis to infant ERPs. Our study showed that the duration of the microstate likely to correspond to attention engagement in response to faces with direct gaze (M4) was one of the strongest predictors of later ASD within the family history group. The strength of this microstate (the scalp field response^51^) was also the microstate feature that contributed the most to the prediction of dimensional variation in social skills at the individual level across the whole sample. Taken together, our results consistently suggest that individual differences in the strength and timing of the brain response to social and non-social stimuli in infancy are associated with later dimensional variation in social skills and categorical ASD outcome.

### Prediction of later categorical and dimensional phenotypes

Whilst our results provide evidence of atypical social attention, there are a number of interesting patterns consistent with emergent evidence that (a) the neurodevelopmental drivers of categorical ASD vs dimensional variation in ASD-relevant domains are different^45,57,58^; and (b) ASD does not represent a homogenous category^59,60^. First, our results raise the intriguing possibility that the timing of a brain response might be more specifically related to ASD outcome than its strength. In the classic ERP analysis, Nc latency was longer in the group of infants who received a diagnosis of ASD at 3 years than in other groups; we observed that the FH-ASD group showed slower responses to the Noise stimulus relative to the face stimuli whilst the other groups showed the opposite pattern (Fig. 3B). Similarly, Jones and colleagues previously found that later ASD outcome was associated with faster offset of the Nc to faces compared to objects at 6 months of age^15^, a result they interpreted as consistent with shallower attention engagement. Further, the data-driven analysis indicated that the duration of all three universally observed microstates in the 300–800 ms time-window in response to faces with direct gaze contributed to prediction of categorical ASD outcome. This may reflect the fact that the latency of a single waveform feature (like the Nc) may index the combined alterations in the timing of a number of underlying processes.

Although the meaning of individual microstates remains to be determined, examining the topoplots and time-courses suggests that Microstate 1 (M1) likely represents the return to a ‘baseline’ brain state; Microstate 4 (M4) occurs in the time-window and with the topography of the Nc, and thus is hypothesised to reflect attention engagement^27,31^; and Microstate 2 (M2) appears to reflect the latter part of the Nc and the onset of the slow wave, possibly linked to cognitive processing of the stimulus independent of the state of attention^30,61^. Specifically, ASD was associated with shorter duration of M1 (return to baseline) and M4 (attention engagement), and longer duration of M2 (cognitive processing) to faces with direct gaze. M2 is characterised by a fronto-central positivity which could represent the beginning of the positive slow wave observed after the Nc component^29,62^, reflecting memory updating and recognition^27^. Increased and prolonged activity in the M2 state could indicate that in infants with emerging difficulties in the social domain, processing resources are becoming devoted to static faces within the same age period (between 6 and 11 months) that typically developing infants start to specialise in more complex social stimuli, such as live scenes^23^. Of note, observing the classic ERP results we see that the FH infants had significantly reduced attention in response to faces with direct gaze compared to the non-social control stimulus, while the noFH-noASD infants showed reduced attention for faces with averted gaze only, as expected based on previous research^25,63^. Delayed neural specialization for face processing, especially in the presence of direct gaze in this critical period might be responsible for the onset of a divergent developmental pathway of behavioural correlates of social attention, such as looking at the eyes and making eye-contact, which might have cascading effects on social learning^11,64^ in children with inherited susceptibility^20^. Mapping trajectories to ASD is likely to require age-sensitive testing paradigms.

Although intriguing, a number of points indicate that there is substantial heterogeneity in this pattern within infants with later ASD. In the data-driven analysis, while the combination of microstate features showed a high specificity for prediction of ASD, sensitivity was weak. This means that some of the children with ASD did not show this profile. Further, the hypothesis-driven classic ERP analysis suggesting Nc latency differences between infants with and without ASD were only marginally statistically significant. It is possible that the subset of children with ASD who were not correctly classified by the genetic algorithm showed atypicalities in timing of processing of the Noise stimulus, given that microstate duration to Noise was not selected amongst the high incidence features for prediction. Recent studies analysing patterns of functional connectivity have also suggested that brain timing might be one of the crucial features of ASD^37,65^, and might be a useful feature for subtyping^66^.

In contrast to neural timing, results for amplitude (or strength) were more consistent with a dimensional relation to social skills and ASD familial liability but not specific to ASD outcome. In the ERP analysis, the FH-noASD infants showed a similar profile of smaller Nc to FD than to Noise as the FH-ASD group. The Nc amplitude findings are consistent with previous research suggesting that social attention may be a trait marker of genetic susceptibility, or endophenotype, of ASD^11,67–69^. Endophenotypes are measures that are closer to the biological bases of a condition than clinical phenotypes^70^; they must be reliably quantifiable, they are observed earlier than clinical symptoms, and they are found to a higher extent in relatives of affected individuals than in the typical population^71^. A weak Nc mean amplitude to faces with direct gaze is promising in this regard, since it represents a direct measure of brain activity; it has been replicated in multiple cohorts^15^; it can be observed at 8 months, that is prior to clear behavioural symptoms; and it is present in FH infants who do not necessarily develop ASD or infants with parents with autistic-like social traits at an intermediate level^69^. Constantino and colleagues recently showed that eye-tracking measures of social attention, which are atypical in toddlers with ASD, are highly heritable^67^; such an approach should now be taken with neural measures.

### Social specificity

Both our analyses are consistent with other evidence that atypicalities relevant to ASD are not limited to social attention^72^. Figure 4B shows that infants with later ASD had overall slower Nc latency for Noise than infants without later ASD, although post-hoc tests did not reach statistical significance. Differences at the dimensional level were also not confined to faces. Across the sample, there were larger mean Nc amplitudes in response to the Noise stimulus than to faces with direct gaze (in line with what observed in similar studies looking at Nc responses to different non-social stimuli^15,31,72^). Possibly, larger Nc amplitude in response to the Noise stimulus could be explained by the fact that our experiment featured faces and Noise stimuli in a 2 to 1 ratio. As the Nc is enhanced for less frequent stimuli^27,61^, this could have increased the amplitude of the Nc. Moreover, infants were unlikely to have encountered a ‘scrambled face’ image before, increasing its novelty^73^. However, other studies found the same pattern of increased amplitude of the Nc in a group of infants with and without a family history of ASD in response to pictures of toys presented in equal proportion to faces^15,31^. Both in our study and in Jones and colleagues’ study, although typical infants showed more or similar interest in visual noise or toys than faces, infants with an older sibling with ASD showed a relatively greater exaggeration of interest to the non-social stimulus. Since attention serves to direct resources in the context of competition^33^, it may be the balance between attention directed towards social and non-social stimuli (rather than either in isolation) that is most relevant to consider.

Indeed, the machine-learning algorithm revealed that a stronger scalp field of M4 in response to Noise added to the prediction of low social skills at 3 years, though it was less predictive than the same microstate response to faces with direct gaze. This may be consistent with eye-tracking studies showing that the ability to disengage attention (applicable to social and non-social contexts) is atypical in infants with a family history of ASD^74,75^ and that a combination of differences in attention style both towards social and non-social stimuli underlie atypical developmental trajectories^39,76–78^. In fact, infants with a familial history of ASD have enhanced visual search abilities^79^, better working memory for non-social stimuli^80^, shorter time intervals between fixations^81^ and difficulties in disengagement during visual orienting^82^. Those characteristics are predictive of more severe ASD symptoms in toddlerhood. Using a data-driven approach to build models that incorporate different types of phenotypes has value for identifying these profiles and will help understand the mechanisms of risk and resilience in the development of social cognition^83^.

### Functional states of the whole brain during attention engagement

The present study illustrates the power of an integrated spatio-temporal analysis of brain activity as a complement to the classic ERP method. A data-driven analysis confirmed that Microstate 4 in response to faces with direct gaze was the most consistently informative of categorical ASD outcome and dimensional variation in social skills. This microstate was characterised by a dipole that presented as a frontal negativity and occipital positivity, likely contributing to both the Nc and the P400 component (M4, Fig. 2A). These two components have both been previously associated with early ASD. Previous eye-tracking and EEG research has indicated that infants with a family history of ASD show atypical attention between 300 and 700 ms after the adult has initiated direct gaze^15,18,25^. At a brain level, face ERP studies have shown that infants with a familial history of ASD might show a different profile of the P400^84^, when attending to faces with direct gaze compared with infants with no family history of ASD. Microstate analysis provides a way to unify ERP signals across scalp regions^32^. Previous studies have argued that, in infants, P400 and Nc are largely generated by the same dipole sources^85^ and highly correlated during attention engagement with static faces^15^. Studying brain states (microstates) underlying attention allows us to recognize how functional processes might be affected by atypical connectivity characteristics in the whole brain^7,49^.

### Limitations and future directions

We showed that brain states reflecting periods of synchronized network activation underlying cognitive processes^86^ can be identified in the infants’ brain. Importantly, although microstates have been widely used to study brain functioning in psychiatric conditions^86^, they have not been used in infancy research. Our approach in this sense is highly novel; replication of the ‘prototypical’ maps estimation is needed. Future work should explore incorporation of other measures to allow machine-learning algorithms to capture multidimensional profiles and obtain stronger predictions of later ASD. Of note, the performance of the classification algorithms was only marginally significant, possibly due to the small sample of the training dataset, therefore, larger samples should be used in the future to produce more robust results. We also acknowledge that in our study nearly 40% of the original sample was excluded from analyses due to insufficient EEG data for an ERP design. The clinical impact of the present findings is somewhat limited by the reduced sample size, especially for the FH-ASD group. In fact, due to the limited sample size features for prediction were selected on the same data that the SVM classification was implemented. An independent replication sample would be needed to exclude spurious conclusions of the classification algorithm.

The study of microstates in infancy allows us to move from looking at static indices to measuring sequences of functional processes in the social brain^87^. Understanding brain states during social interaction is especially relevant for early intervention in children with ASD. In fact, EEG is a non-invasive neuroimaging technique that has been used to assess the effects of intervention in boosting social attention skills^88,89^. Our study revealed the potential that microstate features identified in infants during attention to social stimuli contribute, together with sex-specific differences and developmental level for cognitive abilities measured with the MSEL, to predict later outcome. Interestingly, the contribution of M4 features in response to faces with direct gaze was larger than developmental level at the same age. With optimised methods, microstates could be used to monitor impact and mechanism of interventions targeting social (interaction) processes^90^, or could be used to plan personalised interventions in infants at high vulnerability for atypical neurodevelopmental outcome. Microstates analysis has been successfully used with adults to examine information intake in real time^86,91^. Exploring brain states changes in continuous EEG signal recorded in response to live stimuli is a next, promising avenue to identify optimal windows, and consequently tailor opportunities, for learning in the real world.

In conclusion, our hypothesis-driven analyses of event-related neural activity over the frontal areas converged with data-driven investigations of the spatio-temporal characteristics of the entire brain state, indicating that atypical attention engagement in infancy contributes to differences in social cognition at three years of age. Specifically, we found that the strength of the attentive brain response to faces with direct gaze contributes to the prediction of later dimensional variation in social skills, while the timing of this process might indicate early atypicalities specifically associated with ASD outcome. Future multidisciplinary research is needed to expand the present findings by incorporating multiple measurements for individual prediction of later outcome. Models including measures of genetic liability as well as brain and behavioural responses to social and non-social stimuli obtained at multiple time points within the first years of life will shed light on the biological mechanisms underlying individual differences in developmental trajectories.

## Supplementary information

Supplementary Materials

## Data Availability

Analysis scripts of the present study are available upon reasonable request to the authors.
