# Correction: Attentive brain states in infants with and without later autism

**DOI:** 10.1038/s41398-021-01368-w

**Published:** 2021-05-10

**Authors:** Anna Gui, Giorgia Bussu, Charlotte Tye, Mayada Elsabbagh, Greg Pasco, Tony Charman, Mark H. Johnson, Emily J. H. Jones

**Affiliations:** 1 Centre for Brain and Cognitive Development, Birkbeck College, University of London, Malet Street, London, WC1E 7HX UK; 2 Department of Cognitive Neuroscience, Donders Institute for Brain, Cognition and Behaviour, Radboud University Medical Center, Kapittelweg 29, 6525 EN Nijmegen, The Netherlands; 3 Department of Child & Adolescent Psychiatry & Department of Psychology, King’s College London, De Crespigny Park, London, SE5 8AF UK; 4 Montreal Neurological Institute, McGill University, 3801 Rue University, Montréal, QC H3A 2B4 Canada; 5 Department of Psychology, Institute of Psychiatry, Psychology & Neuroscience, King’s College London, De Crespigny Park, London, SE5 8AF UK; 6 Department of Psychology, Cambridge University, Downing Street, Cambridge, CB2 3EB UK

**Keywords:** Neuroscience, Human behaviour

Correction to: *Translational Psychiatry*

10.1038/s41398-021-01315-9 published online 30 March 2021

The original version of this article unfortunately contained mistakes inserted by the journal after proofing. There was an error in the last column of Table 1. Table 1 reported the Greek letter beta instead of eta.

**Table 1:** ▓.

	noFH-noASD	FH-noASD	FH-ASD		
*N* current study	40	72	19		
Phase (1/2)	31/9	22/50	9/10		
Sex (M/F)	16/24	34/38	15/4		
**Participants**	**Mean (s.d.)** **Min–max**	**Mean (s.d.)** **Min–max**	**Mean (s.d.)** **Min–max**	***p*-value**	${\mathbf{\eta }}_{\boldsymbol{p}}^2$
Age (days)	244.97 (40.65) 6–11	261.33 (36.66) 6–11	251.21 (31.64) 6–10	0.079	0.04
*8 months*					
MSEL Composite Score	106.33 (11.54) 86–132	103.15 (15.09) 70–134	100.17 (16.75) 77–139	0.285	0.02
VABS Composite Score	100.67 (12.74)^a^ 78–130	93.51 (13.53)^a^ 66–150	92.72 (10.83) 71–113	0.015*	0.07
VABS Socialization Score	103.23 (12.78) 81–132	98.85 (12.97) 70–152	98.22 (10.03) 81–118	0.175	0.03
VABS Communication Score	101.88 (13.03)^a^ 66–123	94.75 (16.77)^a^ 55–143	94.84 (11.51) 70–112	0.048*	0.05
VABS Daily Living Skills Score	100.55 (15.25) 54–122	100.79 (13.63) 54–143	97.74 (13.51) 77–117	0.697	0.01
VABS Motor Skills Score	97.45 (14.11)^a,b^ 73–127	85.58 (16.19)^a^ 56–144	84.16 (13.69)^b^ 56–106	<0.001*	0.12
*3 years*					
MSEL Composite Score	115.50 (15.06)^a^ 80–147	108.35 (20.61)^b^ 63–145	92.39 (26.19)^a,b^ 49–142	0.001*	0.12
VABS Composite Score	107.26 (9.17)^a,b^ 93–131	99.06 (9.15)^a,c^ 78–121	82.05 (11.74)^b,c^ 57–100	<0.001*	0.41
VABS Socialization Score	105.79 (7.11)^a,b^ 94–122	99.21 (9.51)^a,c^ 72–116	78.42 (12.49)^b,c^ 61–110	<0.001*	0.47
VABS Communication Score	107.94 (11.05)^a,b^ 85–139	100.89 (10.79)^a,c^ 76–125	87.83 (15.12)^b,c^ 52–112	<0.001*	0.24
VABS Daily Living Skills Score	70.76 (15.86)^a,b^ 27–96	60.54 (20.56)^a,c^ 16–95	27.00 (23.91)^b,c^ 1–90	<0.001*	0.34
VABS Motor Skills Score	101.65 (13.22)^a,b^ 61–124	93.33 (10.93)^a,c^ 70–124	85.26 (10.78)^b,c^ 64–100	<0.001*	0.12
ADOS-2 CSS	2.50 (1.86)^a^ 1–7	2.70 (2.12)^b^ 1–8	5.47 (2.99)^a,b^ 1–10	<0.001*	0.18

*noFH-noASD* no Family History of Autism Spectrum Disorder (ASD) and no diagnosis at 3 years, *FH-noASD* Family History without a diagnosis of ASD at 3 years, *FH-ASD* Family History of ASD who received a diagnosis of ASD at 3 years, *MSEL* Mullen Scales of Early Learning, *VABS* Vineland Adaptive Behavior Scales, *ADOS2-CSS* Autism Diagnostic Observation Schedule, 2nd edition, with Calibrated Severity Scores calculated as explained in SM1.

*N*: number of subjects with available scores; s.d.: standard deviation; *p*-value of the one-way ANOVA with outcome groups as between-subjects factor, for age, MSEL and VABS scores, and Kruskal–Wallis non-parametric test for ADOS scores;

${\mathbf{\eta }}_{\boldsymbol{p}}^2$: partial eta-squared as a measure of the effect size.

^a,b,c^ Superscript letters denote that groups are significantly different from each other based on Tukey’s Honest Significant Difference post-hoc analyses with 95% family-wise confidence level for age, MSEL and VABS scores, and based on pairwise comparisons using Mann–Whitney *U* test with Bonferroni correction for multiple comparisons for ADOS-2 CSS.

*p < 0.05.

In addition, there were errors in the figure legends of figures 3 and 4. Figure 3 should read: Mean Nc features by group and stimulus. Mean amplitude (A) and peak latency (B) of the Nc by stimulus and ASD liability groups, averaged across the frontal regions. All error bars represent ±1 standard error. Figure 4 should read: Relationship between Nc features and later dimensional variation in social adaptive skills. Mean amplitude (A) and peak latency (B) difference between face with direct gaze and Noise at 8 months, on the x-axis. Translational Psychiatry apologizes for the errors. The original article has been corrected.

