# Supplementary material for: Attentive brain states in infants with and without later autism
Source: Transl Psychiatry. 2021 Mar 30;11:196. doi: 10.1038/s41398-021-01315-9 (PMC8009890; doi:10.1038/s41398-021-01315-9)
Supplement: Supplementary file 1 — Supplementary Materials [file 41398_2021_1315_MOESM1_ESM.docx]

**SUPPLEMENTARY MATERIALS**

**Supplementary Methods**

**SM1. Original BASIS sample and behavioural assessment**

A total number of 247 children participated in the British Autism Study of Infant Siblings ([www.basisnetwork.org](http://www.basisnetwork.org)). Fifty-four infants with a family history of Autism Spectrum Disorder (ASD), hereafter FH, as they were younger siblings of children with ASD and 50 with no family history (noFH) were recruited in the initial phase of BASIS, Phase 1^1^. Subsequently, 116 FH and 27 noFH participated in Phase 2. All noFH infants, recruited from a volunteer database at the Birkbeck Centre for Brain and Cognitive Development, had gestational age between 37 and 42 weeks, except one born prior to 37 weeks, and no first or second relatives with ASD.

Between 6 and 11 months of age, infants received a two-days assessment during which a series of measures were collected, such as parent-report questionnaires (including the Vineland Adaptive Behaviour Scales, VABS)^2^, behavioural assessments, eye-tracking experiments and the face/gaze Event-Related Potentials (ERP) task analyzed in the current study. Developmental level of cognitive ability was assessed at each visit using the Mullen Scales of Early Learning (MSEL)^3^, a standardised measure of early non-verbal reasoning, motor and language skills (Table 1 shows scores at 8 and 36 months for the sample included in the present study).

At the age of three, a clinical assessment was provided by an independent team to determine whether the child had developed ASD. 239 children participated in the follow-up visit. Of the 8 children who dropped out, 4 FH (across Phases) were excluded from the current analysis, while 4 noFH children were included in the analysis in the control group. Experienced clinical researchers administered, or closely supervised the administration of, a battery of clinical research measures to the 36-month-old children and determined the clinical outcome by reviewing all the available measures. Among these, the Autism Diagnostic Observation Schedule-Generic^4^ (ADOS-G) is a semi-structured observational assessment used to determine the presence of autistic behaviours and the severity of ASD symptoms. ADOS-2 severity scores, reported in Table 1 in the main text, were calculated by using the relevant raw item scores from the original ADOS-G assessment to re-calculate subscale and total scores as per ADOS-2. Comparison Severity Scores (CSS) were obtained from the new ADOS-2 overall total. Additionally, parents were interviewed using the Autism Diagnostic Interview – Revised (ADI-R)^5^, a detailed interview covering early development and autism diagnostic features, and required to fill the parent-report questionnaire VABS (composite score and subdomains standard scores are reported in Table 1 for children who provided artifact-free EEG data for the current study).

Of the 166 FH infants who participated in BASIS Phase 1 and 2, 34 (20%) met criteria for an ASD diagnosis (FH-ASD) using ICD-10 criteria (Phase 1) or DSM-5 (Phase 2), 88 (53%) were classified as typically developing (FH-TD) and 44 (27%) were identified as showing other signs of atypical development (FH-Other) by scoring above the autism spectrum threshold on the ADOS-G, and/or scoring above the autism threshold on the ADI-R, and/or scoring below –1.5 standard deviation on the MSEL Early Learning Composite, Visual Reception, Receptive Language or Expressive Language subscales.

One hundred-thirty-one infants were included in the current study as they provided sufficient artifact-free EEG and behavioural data. Forty were noFH children while 91 were FH children, who were classified into the three outcome groups following the 3-year visit: 19 were identified as FH-ASD; 48 as FH-TD and 24 as FH-Other. Of the children in the FH-Other group, 14 met ADOS-G criteria only, 3 met both ADOS-G and ADI-R criteria, 3 met both ADOS-G and MSEL criteria, and 4 met MSEL criteria only. In the present study, the FH-TD and FH-Other were collapsed into the FH-noASD group (total n=72), in line with similar research^1,6^. None of the noFH children met criteria for an ASD research diagnosis nor had received a community diagnosis of ASD by 3 years of age, and are therefore hereafter identified as noFH-noASD.

For all 91 children with an older sibling with a community clinical diagnosis of ASD (hereafter probands), parents completed the Development and Well-Being Assessment (DAWBA)^7^ and/or the Social Communication Questionnaire (SCQ)^8^. Seventy-one probands met criteria on both the DAWBA and SCQ (ASD threshold ≥15). Six children scored <15 on the SCQ and one was missing the SCQ, but no exclusions were made due to meeting threshold on the DAWBA and expert opinion. For 12 probands, confirmation of local clinical diagnosis was only available via the SCQ and for one child no questionnaire measures were available. Screening for possible ASD in the older siblings of the 40 noFH-noASD infants was undertaken using the SCQ, with no child scoring above the instrument cut-off for ASD. For one noFH child the SCQ was missing. Medical history review confirmed a lack of ASD within first- and second-degree relatives.

**Table S1** Demographic characteristics and mean scores of behavioural measures for the study participants, collected at the time of EEG testing (8 months of age) and diagnostic assessment (3 years of age). The group of excluded participants (n=116) is compared with the sample for the present study (n=131).

| **Participants** | **Excluded** | **Present Study** |  | |
| --- | --- | --- | --- | --- |
| **Males/Females** | 55/61 | 65/66 |  | |
| **Phase 1/ Phase 2** | 42/74 | 62/69 |  | |
| **N noFH-noASD** | 37 | 40 |  | |
| **N FH-noASD** | 60 | 74 |  | |
| **N FH-ASD** | 15 | 19 |  | |
|  | **M (s.d.)**  **Min - Max** | | **p** | **Cohen’s D** |
| **Age at 8 months** | 8.36 (1.11)  6 - 11 | 7.92 (1.27)  6 - 11 | 0.06 | 0.36 |
| **8 months** | | | | |
| **MSEL Composite Score** | 104.13 (15.69)  64 - 143 | 103.80 (14.42)  70 - 139 | 0.86 | 0.02 |
| **VABS Composite Score** | 95.59 (13.55)  49 - 144 | 95.58 (13.31)  66 - 150 | 1.00 | <0.001 |
| **36 months** | | | | |
| **MSEL Composite Score** | 107.41 (24.54)  49 - 147 | 107.79 (20.99)  49 - 147 | 0.72 | 0.02 |
| **VABS Composite Score** | 98.85 (13.50)  52 - 121 | 98.70 (12.41)  57 - 131 | 0.93 | 0.01 |
| **ADOS2-CSS** | 2.83 (2.39)  1 - 10 | 3.06 (2.41)  1 - 10 | 0.38 | 0.10 |

noFH-noASD: infants without a family history of ASD and without a diagnosis of ASD at three years of age, FH-noASD: infants with a family history of ASD who did not meet criteria for ASD at three years of age, FH-ASD: infants with a family history of ASD who were diagnosed with ASD at three years of age; N: number of subjects; M: mean; s.d.: standard deviation; p: p-value of the independent samples t-test comparing the group of excluded participants with the sample for the present study, Cohen’s D: measure of the effect size. MSEL: Mullen Scales of Early Learning; VABS: Vineland Adaptive Behavior Scales; ADOS: Autism Diagnostic Observation Schedule, 2^nd^ edition, with Calibrated Severity Scores, calculated as explained in SM1.

**Figure S1** Diagram showing the number of participants initially recruited as part of the British Autism Study of Infant Siblings (original BASIS sample, top cell) and reasons for subsequent exclusion, leading to the actual sample for the current study (bottom cell). Orange cells contain information on the number of participants who were excluded at various steps.

**SM2. EEG data pre-processing**

EEG data were collected while 50 trials were presented continuously for as long as the child remained attentive. Stimuli were colour pictures of a female model whose gaze was directed either toward (Face with Direct Gaze or FD, Figure S2.A) or away (Face with Averted Gaze or FA, Figure S2.B) from the infant, and a control stimulus, constructed by randomizing the phase spectra of the face stimulus while keeping the amplitude and colour spectra constant (Figure S2.C). EEG data were digitized with a sampling rate of 500 Hz and band-pass filtered between 0.1-100 Hz. The vertex has been used as reference (Cz in the conventional 10/20 system). Data were stored and analyzed offline in EGI NetStation 4 (for Phase 1) and 5 (for Phase 2) using the same protocol as in Elsabbagh et al.^1^ (which included the Phase 1 participants to the present study). The EEG recording was segmented into 1000 ms (-200 to 800 ms peri-stimulus window for the FD condition, -200ms to 795 ms for FA and Noise).

Video-coding procedure was used to exclude those segments where the infant displayed gaze shifts, looked away from the screen or was crying during or 100 ms before stimulus presentation. Valid data were re-segmented and baseline corrected, with baseline from -195 ms till the stimulus onset. Segments with significant artifact were identified and removed through automatic detection. Specifically, for each segment, channels with EEG signal >400 μV were removed as bad channels; continuous data where the signal reached amplitudes >400 μV for 1000 ms were removed as likely representing eye-blinks and for 160 samples were removed as eye movements. Channels were marked as bad if more than 75% of the data was detected as artifact. Following this automatic procedure, individual trials were visually inspected by experienced EEG researchers (M.E, C.T.) and further any channels showing artifacts were excluded. Single trials were excluded if they had more than 12 bad channels, while missing data from 12 or fewer channels were interpolated. Infants were excluded if there were less than 10 minimal-artifact trials in any condition. Data were then re-referenced to the average. For each participant with good data obtained for a minimum of 10 trials per condition, stimulus-locked epochs were averaged for the following conditions: FD, FA, Noise. Table S2 shows the mean number of valid trials per group for each condition.

**C**

**A**

**B**


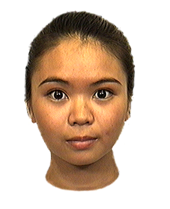

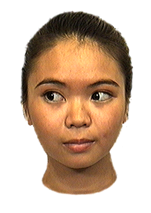

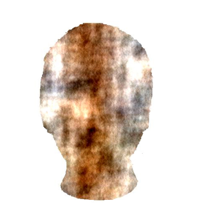


**Figure S2** Experimental stimuli for the EEG task. **A** Face with Direct Gaze, **B** Face with Averted Gaze, **C** Noise.

**Table S2** Number of valid trials from the EEG recording per condition (Face Direct Gaze, Noise, Face Averted Gaze), for each ASD liability group (noFH-noASD, FH-noASD, FH-ASD). P-values and effect sizes of one-way ANOVAs comparing the number valid trials between outcome groups for each condition are reported.

| **Condition** | **Outcome Group** | **N** | **Mean trials** | **s.d.** | **p** | $\boldsymbol{\eta}_{\boldsymbol{p}}^{\boldsymbol{2}}$ |
| --- | --- | --- | --- | --- | --- | --- |
| **Face Direct Gaze** | noFH-noASD | 40 | 19.925 | 6.290 | 0.148 | 0.029 |
|  | FH-noASD | 72 | 17.97 | 5.721 |  |  |
|  | FH-ASD | 19 | 20.368 | 7.166 |  |  |
| **Noise** | noFH-noASD | 40 | 26.450 | 8.019 | 0.554 | 0.009 |
|  | FH-noASD | 72 | 25.569 | 7.928 |  |  |
|  | FH-ASD | 19 | 27.789 | 9.247 |  |  |
| **Face Averted Gaze** | noFH-noASD | 40 | 20.175 | 6.425 | 0.235 | 0.022 |
|  | FH-noASD | 72 | 18.361 | 5.673 |  |  |
|  | FH-ASD | 19 | 20.421 | 8.221 |  |  |

noFH-noASD: infants without a family history of ASD and without a diagnosis of ASD at three years of age, FH-noASD: infants with a family history of ASD who did not meet criteria for ASD at three years of age, FH-ASD: infants with a family history of ASD who were diagnosed with ASD at three years of age; N: number of subjects; Mean trials: mean number of valid trials; s.d.: standard deviation; p-value: p-value of the one-way ANOVA with outcome groups as a between-subjects factor,$\boldsymbol{\eta}_{\boldsymbol{p}}^{\mathbf{2}}$: partial eta-squared.

**A**

**C**

**B**


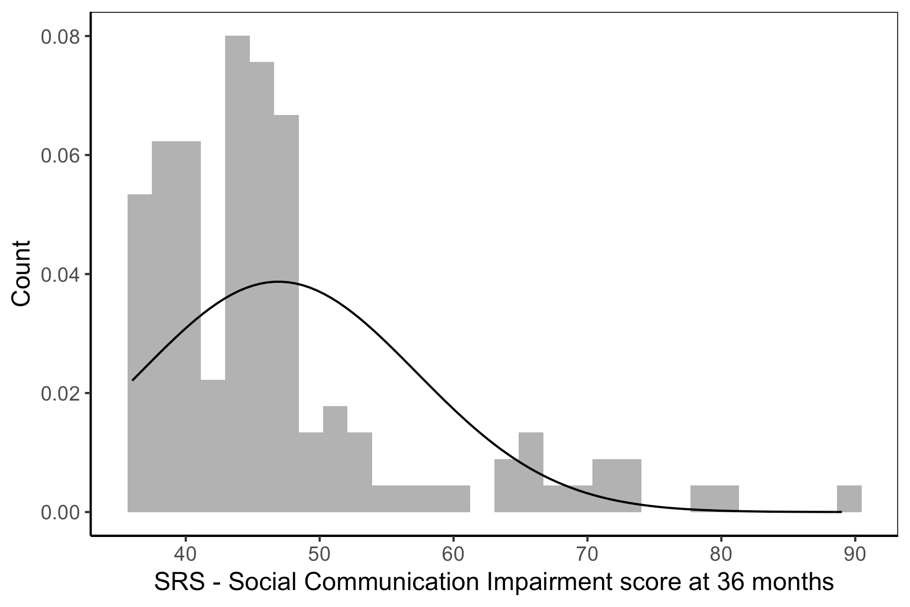

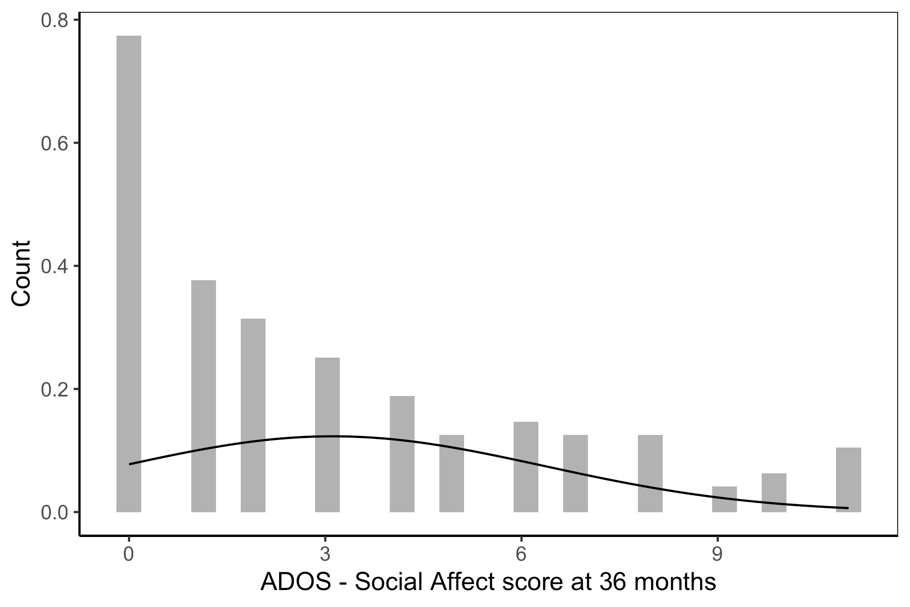

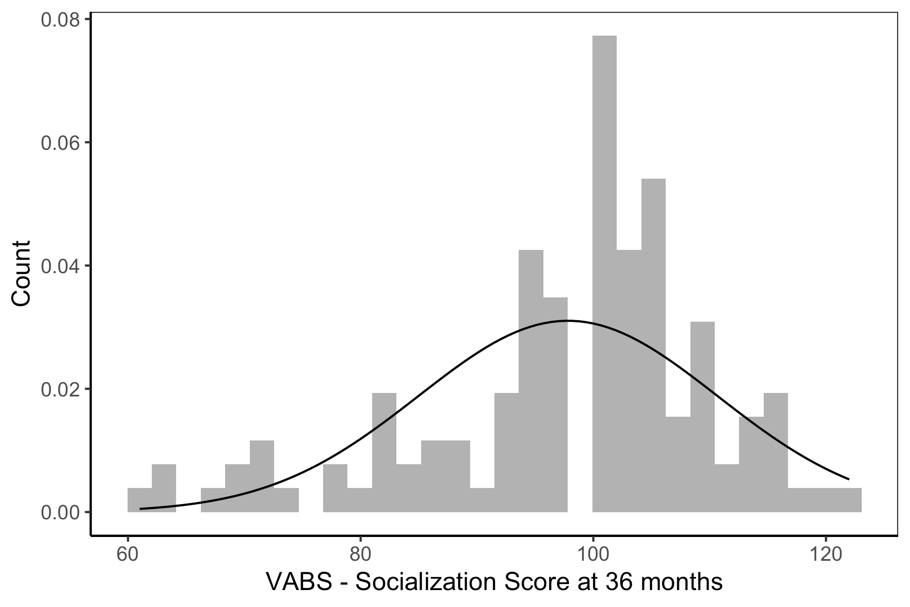


**Figure S3** Distribution of dimensional measures of social skills at three years for the study sample. **A** Vineland Adaptive Behavior Scales (VABS), n=123 – Socialization standard score; **B** Autism Diagnostic Observation Schedule (ADOS), n=126 – Social Affect Calibrated Severity Score; **C** Social Responsiveness Scale (SRS) – Social Communication Impairment t-score, n=123.

**Figure S4** Left (blue), central (red) and right (yellow) frontal electrodes selected based on previous research investigating the Nc component in response to social stimuli in infants^9^.

**SM3. Microstates identification**

Microstate analysis tracks the changes of brain functional states, defined by specific distributions of simultaneously active brain regions, by identifying periods of stable scalp electrical potential topographies recorded by electrodes placed over on the entire scalp^10^. This has been extensively done in the adults’ literature by assigning each time period of the ERP to exactly one of the spatially defined microstate templates obtained from clustering the data into predetermined numbers of topographies (microstate maps or prototypes)^11,12^.

In the present study, the program RAndomization Graphical User interface (RAGU)^13^ was used to identify the optimal number of microstate maps in the group of noFH-noASD infants in the 800 ms following the presentation of the Face with Direct Gaze stimulus. The entire procedure is summarized in the Figure S5. First, ERPs data were re-filtered between 2 and 20 Hz. Cross-validation was applied 250 times, each time randomly splitting the noFH-noASD sample into 20 training datasets and 20 test datasets. Between 1 and 10 microstate classes were estimated using an AACH algorithm. Briefly occurring microstates were suppressed using a segmentation smoothing algorithm^11^, with penalty term for non-smoothness of 0.3 and a window-size for smoothing of 10^12^. The mean correlation coefficient representing the amount of explained variance in the test set (around 0.7) is comparable to values obtained with the same technique for estimation of microstates from on adults’ ERPs data^12^.

A general linear model ('glm' function of the ‘stats’ R-package) was used to test whether changing the number of microstate maps, constructed with the training datasets, significantly increased the amount of explained variance in the test datasets across 250 cross-validation runs (F(9,2490)=5621, p<0.001). Bonferroni-corrected one-tailed pairwise t-tests revealed that the amount of explained variance, initially significantly increasing with increasing number of microstates, did not significantly increase when using models with more four maps (Figure S6, Table S3).

The four “prototypical” maps, reflecting brain states of noFH-noASD infants to the FD, were identified in the individual ERP data for the entire sample in the FD, FA and Noise conditions, between 300 and 794 ms, corresponding to the Nc time window. Of note, as FA and Noise segments ended 795 ms after the stimulus onset, the period between 300 and 794 ms, instead of 800, was chosen for this analysis, given that for the microstate analyses all individual recordings should include the same number of samples.

Microstate features such as duration and mean Global Field Power (GFP) were extracted by RAGU for each map in each of the three conditions (FD, FA and Noise) and entered in the machine-learning analyses.


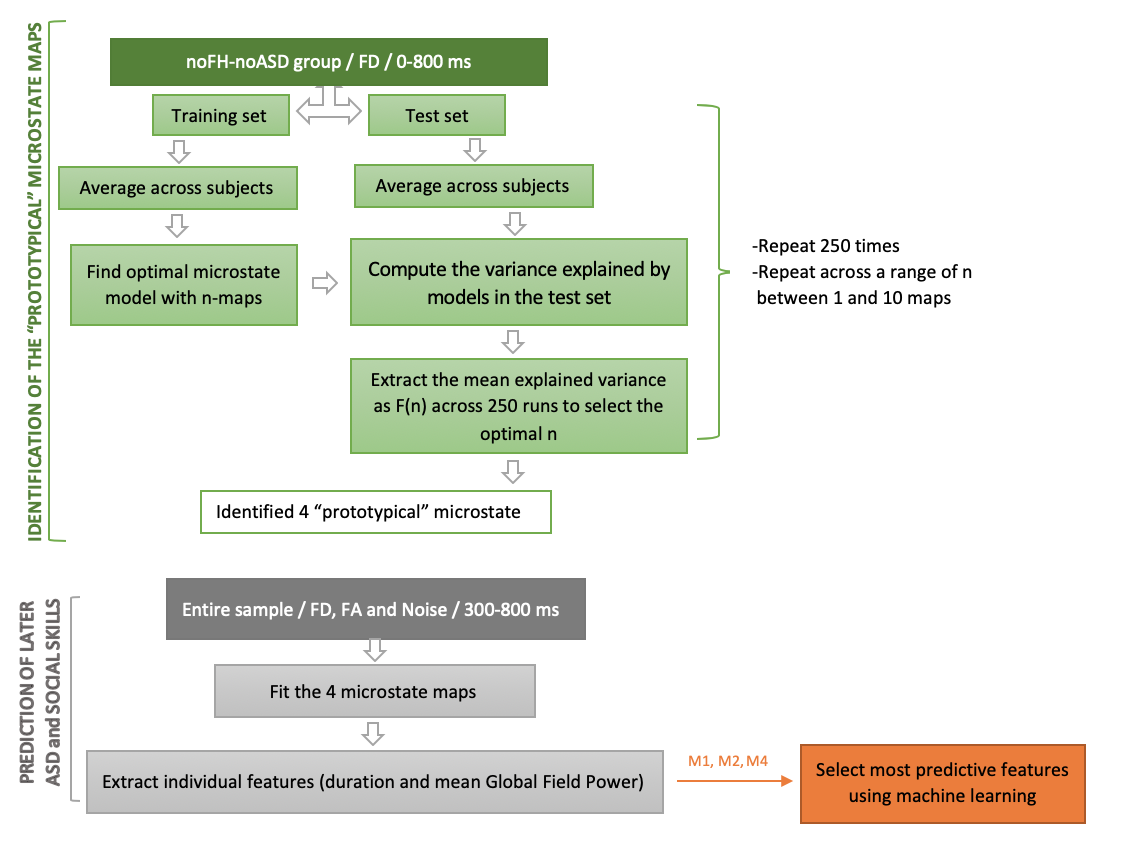


**Figure S5** Flow-chart illustrating the procedure for the microstate analysis. The first part (in green) is adapted from Koenig at al., 2014^12^.

noFH-noASD: infants without a family history of Autism Spectrum Disorder (ASD) and without a diagnosis of ASD at three years of age, FD: Face with Direct Gaze, FA: Face with Averted Gaze.

**
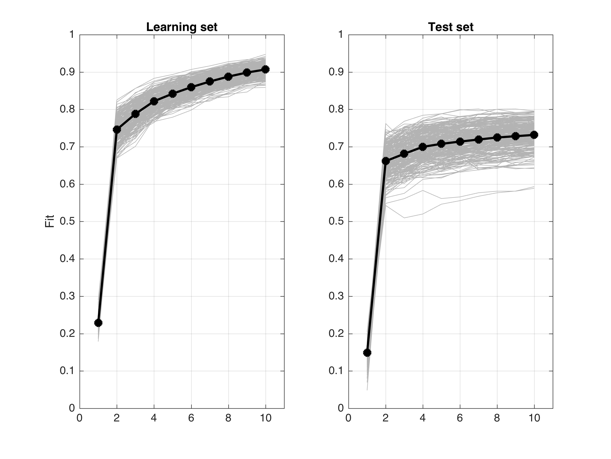
**

**Figure S6** Variance in the scalp field potentials explained by the microstate maps estimated from the learning set (on the left) and tested on the test set (on the right). On the x-axis, the number of microstate maps used to fit the data is displayed. Each of the grey lines represents the performance of the models estimated from one of the 250 randomly created learning sets. The black solid line represents the mean variance explained across 250 cross-validation runs.

**Table S3** Results of the pairwise t-tests to select the optimal number of “prototypical” microstate maps showing that a significantly higher amount of variance in the ERP data in the test set across 250 cross-validation runs was obtained with four microstate maps.

| Microstate models | Exp. Var. mean | s.e. | comparisons  N of maps | Adj. p |
| --- | --- | --- | --- | --- |
| 1 microstate map | 0.149 | 0.002 |  |  |
| 2 microstate maps | 0.662 | 0.003 | 2>1 | <0.0001* |
| 3 microstate maps | 0.681 | 0.003 | 3>2 | <0.0001* |
| 4 microstate maps | 0.700 | 0.003 | 4>3 | <0.0001* |
| 5 microstate maps | 0.708 | 0.003 | 5>4 | 0.382 |
| 6 microstate maps | 0.714 | 0.003 | 6>5 | 1.000 |
| 7 microstate maps | 0.719 | 0.003 | 7>6 | 1.000 |
| 8 microstate maps | 0.724 | 0.003 | 8>7 | 1.000 |
| 9 microstate maps | 0.728 | 0.003 | 9>8 | 1.000 |
| 10 microstate maps | 0.731 | 0.003 | 10>9 | 1.000 |

Exp.Var mean: mean proportion of explained variance of the ERPs data in the test dataset over 250 repetitions; s.e.: standard error; comparisons N of maps: number of microstate maps compared in the post-hoc pairwise one-tailed t-tests; Adj. p-value: Bonferroni-corrected p-values of the indicated contrast.

* p<0.05.

**SM4. Machine learning for individual level prediction**

1. *Prediction of ASD*

The main aims of this analysis were to classify among FH infants those who will receive a diagnosis of ASD at 36 months vs non-ASD siblings using EEG microstates, and to identify the most relevant microstate features for prediction of ASD at 36 months of age. The final sample for classification comprised a total of 91 FH siblings, which was split into a training (70% of the initial sample, n=64) and a holdout validation set (30% of the initial sample, n=27), with the sample partitioning stratified for binary outcome (i.e. FH-ASD vs FH-noASD). First, we performed feature selection on the training set using a genetic algorithm based on a support vector machine (SVM) classifier to extract information about the most relevant features for prediction of ASD; second, we performed SVM classification of FH-ASD vs FH-noASD on the holdout validation set to test final classification performance.

Genetic Algorithm for Feature Selection: The genetic algorithm is a stochastic method for function optimization inspired by the evolutionary process of natural selection on genotype, but it does not necessarily involve genetic data and can be applied to any kind of features. Starting from a collection (population) of candidate solutions (sets of features) built from the available measures, the evolutionary process begins generating successive populations (generations) through mating, crossover and mutation^14^. The fitness is computed for each candidate solution in each generation, and candidate solutions are selected for the recombination pool-based on their fitness value. Overall, selection is based on the Darwinian principle of survival of the fittest, ultimately leading to the best solution for the search problem.

In the present study, fitness was measured by the Area Under the Curve (AUC) of a 10-fold cross-validated SVM classifier built on the set of features under evaluation. The AUC is a measure of predictive accuracy for the model, computed as the area under the Receiver Operating Characteristic (ROC) curve, where the ROC curve is a plot of true positive rate vs false positive rate for the model under evaluation. Population size (n=100) and number of generations (n=200) were selected based on previous experience, while the length of feature sets was selected based on the AUC level reached during the evolutionary process and stability of the process. Once selected the length (n=10), the evolutionary process was repeated 200 times to investigate variability in the feature space. Specifically, the basic steps of the genetic algorithm were:

1) select a couple of feature sets (parents) from the mating pool (initial randomly selected feature sets, population) proportional to their fitness (AUC);

2) shuffle the mating pool randomly;

3) for each pair apply crossover with probability of crossover (p_c_), or else copy parents. Crossover consists in: passing common features between two parents (intersection) to the offspring, select a random crossover point and exchange the information (non-common features between parents) between the two parents.

4) for each offspring (resulting feature set) apply mutation. Mutation consists in a bit-flip with probability p_m_ independently for each bit (each feature in the feature set);

5) replace the initial population with the offspring population.

The feature set providing the highest AUC in the evolutionary process was selected as input for the subsequent classifier analysis (optimal set; see Table S6). Furthermore, the candidate solutions with highest AUC (higher than 75%) were selected and used for frequency analysis on the selected features. In fact, different evolutionary runs may result in different optimal sets of features for the classifier that complement each other and have nearly the same quality for classification, but the incidence of each feature in the evolutionary process provides an estimate of the relevance for the specific classification problem. The features with highest incidence (higher than 80%) were selected as input for subsequent classifier analysis (highest incidence set; see Table S6).

Classifier Analysis: For classification, we used a Support Vector Machine (SVM) algorithm with linear kernel. After feature selection performed by the genetic algorithm (see above), we built 2 classifiers on different input sets of features: (1) the optimal set from feature selection; (2) the set of features with highest incidence (f>0.8) in the feature sets with highest performance (AUC>0.75) during repeated evolution of the genetic algorithm. The classifiers were tested on the holdout validation set. All classification analyses were completed using custom scripts implemented on Matlab R2016b (MATLAB 9.1, The MathWorks Inc., Natick, MA, 2016), and the LIBSVM toolbox^15^ was used for the SVM algorithm. To evaluate classification performance, we computed AUC, sensitivity, specificity, accuracy, negative predictive power (NPV), and positive predictive power (PPV) from the ROC curve. 95% confidence intervals (CI) for each performance metric were computed using bootstrap with 10,000 repetitions. The final metrics with errors were obtained from the average and standard deviation values over 1,000 repetitions of the entire procedure, and the 95% CI of each metric was also averaged over repetitions. We tested for significant difference of the classifier performance (AUC) from chance level through a shuffle test^16^. Labels in the training set were randomly shuffled, and classifiers trained to predict the shuffled random labels. Then, AUC was computed for these classifiers predicting true test labels. This procedure was repeated 10,000 times to estimate the null distribution of AUC and test whether classifiers perform significantly better than random. The p-value of AUC for each classifier is reported. Finally, the same method was used to test differences in performance of the two different classifiers.

1. *Prediction of VABS Socialization scores*

The main aim of this analysis was to identify the most relevant microstate features for prediction of dimensional variation in social skills at three years of age at the individual level. The outcome variable was VABS Socialization scores at three years of age. For prediction, we chose regression with elastic-net regularization to be able to select relevant predictors. Elastic-net is a shrinkage method^17^, namely the estimated coefficients are biased to be small by the addition of a penalty term to the objective function. Compared to other regularization techniques, like lasso or ridge regression, it performs better on data with highly correlated predictors^17^.

The VABS Socialization score was power transformed (level=4) to make it normally distributed (Shapiro W=0.98, p=0.18). As pre-processing, skewness of predictors was checked to be lower than 0.7 and none of them needed to be transformed. Predicting and outcome variables were standardised before being included in the regression model. Leave-one-out cross-validation was used to cross-validate the predictive model, and nested 10-fold cross-validation with 10 repetitions was used for parameter optimization based on minimization of the root mean squared error (RMSE). Analyses were performed using the ‘glmnet’ package in R^18^.

**Supplementary Results**

**Event-related Potentials**

**Table S4** Results of the linear mixed model for Nc mean amplitude.

| Nc mean amplitude | **d.f.** | **AIC** | **BIC** | **logLik** | **χ^2^** | **p** |
| --- | --- | --- | --- | --- | --- | --- |
| **Baseline** | 5 | 7331.261 | 7356.585 | -3660.631 |  |  |
| **Age** | 6 | 7306.067 | 7336.456 | -3647.034 | 27.194 | <.001* |
| **Sex** | 7 | 7308.065 | 7343.519 | -3647.033 | 0.002 | 0.966 |
| **Developmental Level** | 8 | 7308.148 | 7348.666 | -3646.074 | 1.917 | 0.166 |
| **Region** | 10 | 7307.665 | 7358.313 | -3643.833 | 4.483 | 0.106 |
| **Group** | 12 | 7310.746 | 7371.523 | -3643.373 | 0.919 | 0.632 |
| **Stimulus** | 14 | 7283.864 | 7354.77 | -3627.932 | 30.883 | <.001* |
| **Group x Stimulus** | 18 | 7281.618 | 7372.784 | -3622.809 | 10.245 | 0.037* |

d.f.: degrees of freedom,AIC: Akaike information criterion; BIC: Bayesian information criterion; Log.Lik.: log likelihood; χ^2^: chi-square statistic; p: p-value

* p<0.05.

**Table S5** Results of the linear mixed model for Nc peak latency.

| Nc peak latency | **d.f.** | **AIC** | **BIC** | **logLik** | **χ^2^** | **p** |
| --- | --- | --- | --- | --- | --- | --- |
| **Baseline** | 5 | 14509.81 | 14535.13 | -7249.904 |  |  |
| **Age** | 6 | 14510.13 | 14540.51 | -7249.063 | 1.682 | 0.195 |
| **Sex** | 7 | 14509.57 | 14545.02 | -7247.784 | 2.557 | 0.110 |
| **Developmental Level** | 8 | 14511.48 | 14552 | -7247.738 | 0.092 | 0.762 |
| **Region** | 10 | 14507.59 | 14558.24 | -7243.796 | 7.885 | 0.019* |
| **Group** | 12 | 14504.77 | 14565.55 | -7240.384 | 6.824 | 0.033* |
| **Stimulus** | 14 | 14505.33 | 14576.23 | -7238.662 | 3.443 | 0.179 |
| **Group x Stimulus** | 18 | 14504.1 | 14595.27 | -7234.05 | 9.224 | 0.056 |

d.f.: degrees of freedom,AIC: Akaike information criterion; BIC: Bayesian information criterion; Log.Lik.: log likelihood; χ^2^: chi-square statistic; p: p-value.

* p<0.05.

**
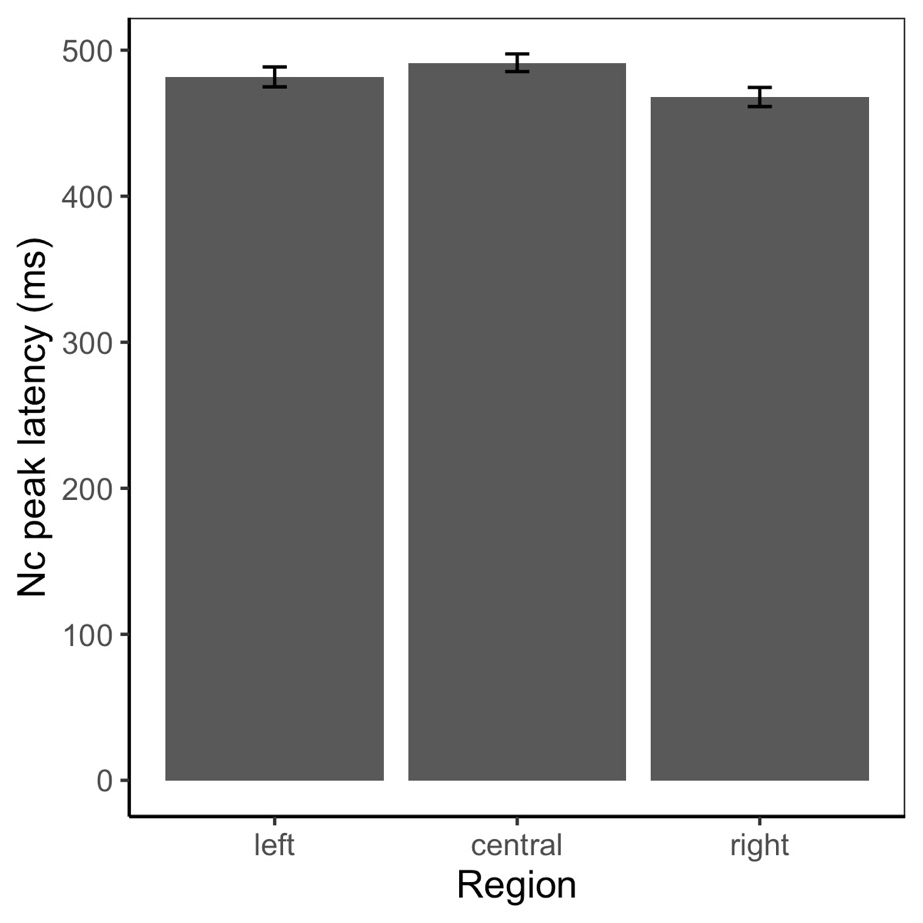
**

**Figure S7** Barplots representing mean peak latency values by region. Error bars indicate ±1 s.e.m..

**Microstates**

**Table S6** Classification performance metrics of classifiers for different input sets of features discriminating infants with an ASD family history who received a diagnosis of ASD (FH-ASD) from those who did not (FH-noASD). The significance of classification AUC was determined by permutation test, the resulting p-values are reported. All metrics are reported as mean [95% bootstrap confidence interval].

|  | Optimal Set | | Highest Incidence | |
| --- | --- | --- | --- | --- |
|  | **Mean** | **p** | **Mean** | **p** |
| AUC | 68.2  [50.9; 94.6] | 0.11 | 62.7  [50.9; 90.0] | 0.09 |
| Accuracy | 70.9  [62.7; 93.2] | 0.29 | 70.0  [63.6; 90.0] | 0.001* |
| Sensitivity | 60.0  [40.0; 100] | 0.58 | 40.0  [40.0; 100] | 0.54 |
| Specificity | 81.8  [36.4; 100] | 0.23 | 100  [31.8; 100] | <0.001* |
| PPV | 76.7  [59.5; 100] | 0.28 | 100  [59.5; 100] | <0.001* |
| NPV | 67.2  [60.2; 100] | 0.57 | 62.5  [62.5; 100] | 0.54 |

AUC = area under the curve; PPV = positive predictive power; NPV = negative predictive power.

Optimal set: sex; duration of microstates M1, M2 and M4 to Face with Direct Gaze (FD); global field power of M4 to FD; duration of M1 and M4 to Face with Averted Gaze (FA); global field power of M1 to FA; duration of M1 and M4 to Noise;

Highest incidence set: duration of M1, M2 and M4 to FD.

* p<0.05

**
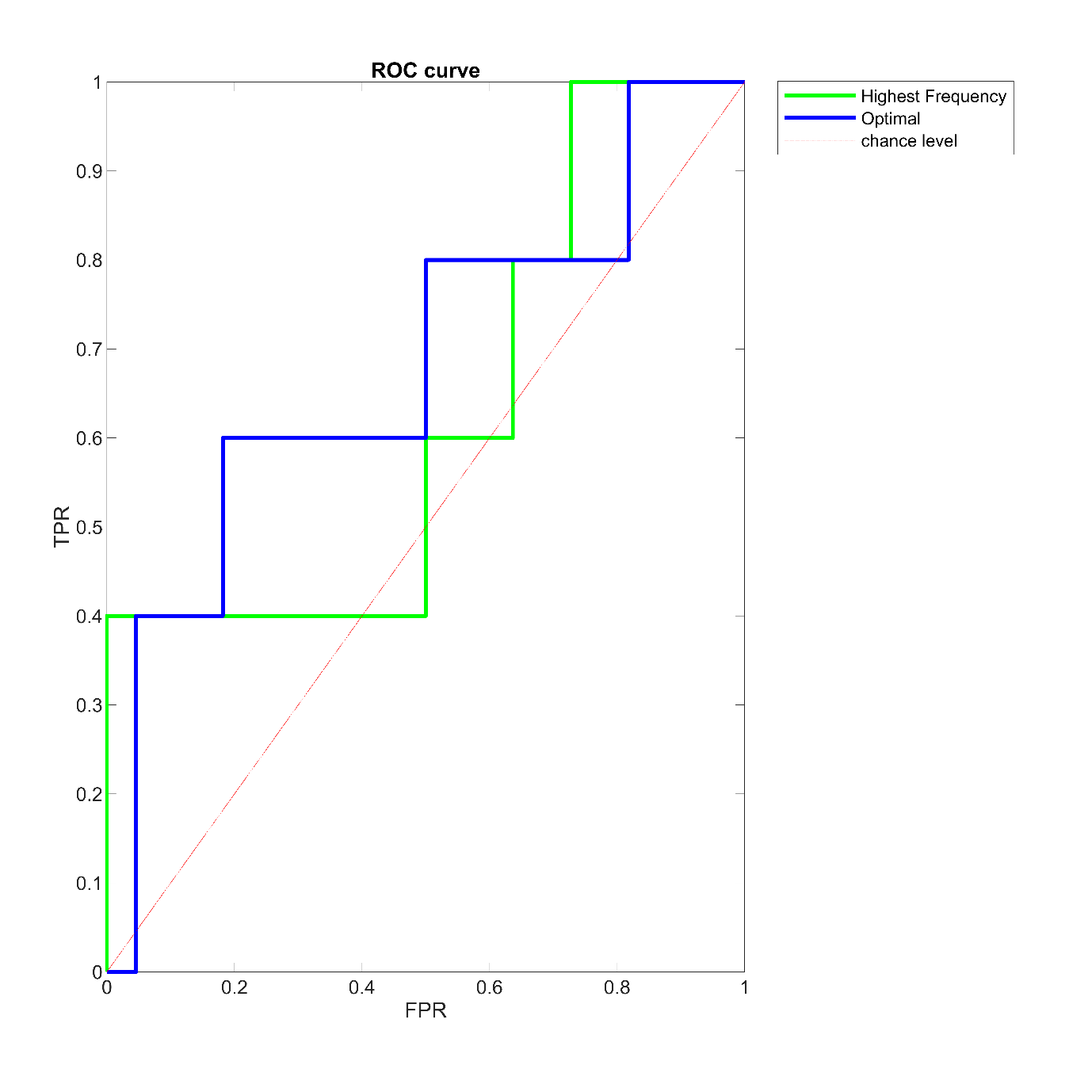
**

**Figure S8** Predictive performance for classification of FH-ASD vs FH-noASD. This figure shows the Receiver Operating Characteristic (ROC) curve for classifiers using different sets of features to classify FH-ASD among the FH infants. Random predictors result in bisecting lines as ROC curves (red dashed line), while deviations in the upper hemifield indicate an increase in predictive accuracy.

**Supplementary References**

1. Elsabbagh, M. *et al.* Infant neural sensitivity to dynamic eye gaze is associated with later emerging autism. *Curr. Biol.* **22**, 338–342 (2012).

2. Sparrow, S. S., Cicchetti, D. V. & Balla, D. A. *Vineland Adaptive Behavior Scales: Second edition (Vineland-2)*. (2005).

3. Mullen, E. M. *Mullen scales of early learning*. (Circles Pines, MN:AGS, 1995).

4. Lord C. *et al.* The Autism Diagnostic Schedule – Generic: A standard measures of social and communication deficits associated with the spectrum of autism. *J. Autism Dev. Disord.* **30**, 205–223 (2000).

5. Lord C., Rutter, M. & Le Couteur, A. Autism Diagnostic Interview-Revised. *J. Autism Dev. Disord.* **24**, 659–85 (1994).

6. Jones, E. J. H. *et al.* Reduced engagement with social stimuli in 6-month-old infants with later autism spectrum disorder: A longitudinal prospective study of infants at high familial risk. *J. Neurodev. Disord.* **8**, 7 (2016).

7. Goodman, R., Ford, T., Richards, H., Gatward, R. & Meltzer, H. The Development and Well-Being Assessment: Description and initial validation of an integrated assessement of child and adolescent psychopathology. *J. Child Psychol. Psychiatry Allied Discip.* **41**, 645–655 (2000).

8. Rutter, M., Bailey, A. & Lord C. *The Social Communication Questionnaire*. (1993).

9. Webb, S. J. *et al.* Developmental change in the ERP responses to familiar faces in toddlers with Autism Spectrum Disorders versus typical development. *Child Dev.* **82**, 1868–1886 (2011).

10. Michel, C. M., Koenig, T., Brandeis, D., Gianotti, L. R. R. & Wackermann, J. *Electrical Neuroimaging*. (Cambridge Univeristy Press, 2009).

11. Pascual-Marqui, R. D., Michel, C. M. & Lehmann, D. Segmentation of brain electrical activity into microstates: model estimation and validation. *IEEE Trans. Biomed. Eng.* **42**, 1–24 (1995).

12. Koenig, T., Stein, M., Grieder, M. & Kottlow, M. A tutorial on data-driven methods for statistically assessing ERP topographies. *Brain Topogr.* **27**, 72–83 (2014).

13. Koenig, T., Kottlow, M., Stein, M. & Melie-García, L. Ragu: A free tool for the analysis of EEG and MEG event-related scalp field data using global randomization statistics. *Comput. Intell. Neurosci.* **2011**, 1–14 (2011).

14. Bäck, T. Evolution strategies: An alternative evolutionary algorithm. *Lect. Notes Comput. Sci.* **1063**, 3–20 (1996).

15. Chang, C. & Lin, C. LIBSVM : A Library for Support Vector Machines. *ACM Trans. Intell. Syst. Technol.* **2**, 1–39 (2013).

16. Golland, P., Liang, F., Mukherjee, S. & Panchenko, D. Permutation Tests for Classification. *Inf Process Med Imaging* **18**, 501–515 (2003).

17. Zou, H. & Hastie, T. Regularization and variable selection via the elastic-net. *J. R. Stat. Soc.* **67**, 301–320 (2005).

18. Friedman, J., Hastie, T. & Tibshirani, R. Regularization Paths for Generalized Linear Models via Coordinate Descent Jerome. *J. Stat. Softw.* **33**, 1–22 (2010).
